# Assessing the Authenticity and Quality of Paprika (*Capsicum annuum*) and Cinnamon (*Cinnamomum* spp.) in the Slovenian Market: A Multi-Analytical and Chemometric Approach

**DOI:** 10.3390/foods14132323

**Published:** 2025-06-30

**Authors:** Sabina Primožič, Cathrine Terro, Lidija Strojnik, Nataša Šegatin, Nataša Poklar Ulrih, Nives Ogrinc

**Affiliations:** 1 Department of Food Science and Technology, Biotechnical Faculty, University of Ljubljana, Jamnikarjeva 101, 1000 Ljubljana, Slovenia; sabina.primozic@hotmail.com (S.P.); natasa.segatin@bf.uni-lj.si (N.Š.); natasa.poklar@bf.uni-lj.si (N.P.U.); 2 Department of Environmental Sciences, Jožef Stefan Institute, Jamova 39, 1000 Ljubljana, Slovenia; cathrine.terro@ijs.si (C.T.); lidija.strojnik@ijs.si (L.S.); 3 Jožef Stefan International Postgraduate School, Jamova 39, 1000 Ljubljana, Slovenia

**Keywords:** paprika, cinnamon, stable isotopes, elemental composition, authentication, traceability, geographical origin, FTIR spectroscopy, phenolics, antioxidant activity

## Abstract

The authentication of high-value spices such as paprika and cinnamon is critical due to increasing food fraud. This study explored the potential of a multi-analytical approach, combined with chemometric tools, to differentiate 45 paprika and 46 cinnamon samples from the Slovenian market based on their geographic origin, production methods, and possible adulteration. The applied techniques included stable isotope ratio analysis (*δ*^13^C, *δ*^15^N, *δ*^34^S), multi-elemental profiling, FTIR, and antioxidant compound analysis. Distinct isotopic and elemental markers (e.g., *δ*^13^C, *δ*^34^S, Rb, Cs, V, Fe, Al) contributed to classification by geographic origin, with preliminary classification accuracies of 90% for paprika (Hungary, Serbia, Spain) and 89% for cinnamon (Sri Lanka, Madagascar, Indonesia). Organic paprika samples showed higher values of *δ*^15^N, *δ*^34^S, and Zn, whereas conventional ones had more Na, Al, V, and Cr. For cinnamon, a 95% discrimination accuracy was achieved between production practice using *δ*^34^S and Ba, as well as As, Rb, Na, *δ*^13^C, S, Mg, Fe, V, Al, and Cu. FTIR differentiated Ceylon from cassia cinnamon and suggested possible paprika adulteration, as indicated by spectral features consistent with oleoresin removal or azo dye addition, although further verification is required. Antioxidant profiling supported quality assessment, although the high antioxidant activity in cassia cinnamon may reflect non-phenolic contributors. Overall, the results demonstrate the promising potential of the applied analytical techniques to support spice authentication. However, further studies on larger, more balanced datasets are essential to validate and generalize these findings.

## 1. Introduction

Ensuring the authenticity of spices is an urgent challenge in global food safety and trade, as increasing rates of adulteration threaten consumer trust, public health, and the integrity of international markets. In the European Union, routine testing has revealed significant fraud rates: 6% for paprika, 11% for turmeric, 14% for cumin, and 48% for oregano [1]. The complexity of global supply chains and the growing demand for organic and natural products further complicates efforts to guarantee authenticity and traceability.

Culinary spices such as paprika (*Capsicum annuum*) and cinnamon (*Cinnamomum* spp.) are indispensable to global cuisines, being valued for their distinctive flavors, vibrant colors, and bioactive compounds, which provide notable health benefits. These functional properties make them highly sought after in the food, pharmaceutical, and cosmetics industries, where they are utilized for their antioxidant, antimicrobial, and anti-inflammatory effects [2,3,4]. Paprika, produced from dried and ground bell peppers, is a staple ingredient in many culinary traditions, particularly in Europe. Although native to Central and South America, paprika is now primarily cultivated in Hungary, Spain, Turkey, Croatia, Serbia, and North Macedonia to meet international demand for both sweet and hot varieties. It grows best in warm, humid climates, which help develop its distinctive color and aroma, but in cooler areas, it must be planted each year because it cannot survive frost [5,6].

Similarly, cinnamon is derived from the bark of evergreen trees of the *Lauraceae* family, which flourish in tropical and subtropical climates where soil and environmental conditions significantly affect its chemical composition and quality [7]. The global cinnamon market is dominated by China, Indonesia, Vietnam, and Sri Lanka, with the latter recognized as the primary producer of the highly valued Ceylon cinnamon (*Cinnamomum verum*), renowned for its delicate flavor and lighter bark. This premium variety is distinguished from the more widely available and lower-cost cassia types (*C. cassia*, *C. aromaticum*), contributing significantly to global trade. The health benefits of cinnamon are attributed to its bioactive constituents, including cinnamaldehyde, eugenol, and cinnamic acid [7].

Given their high economic value, spices like paprika and cinnamon are particularly vulnerable to food fraud, including adulteration with fillers or dyes and mislabeling of origin or species. Such practices pose health risks and undermine consumer trust [8]. Paprika is often subjected to fraudulent practices, such as dilution with inferior materials (e.g., white pepper, curcuma, brick powder), misrepresentation of its geographic origin, and the illegal addition of synthetic dyes like Sudan I and IV to enhance its color [9,10]. The natural degradation of paprika’s color during storage, especially in powdered form, further complicates fraud detection, as increased surface area accelerates oxidation and color loss. Similarly, cinnamon is often adulterated by substituting true Ceylon cinnamon with less expensive cassia bark. Cassia naturally contains significantly higher levels of coumarin, which can exceed safety limits and pose health risks with regular consumption [11,12]. Other common adulteration methods include mixing in fillers such as coffee husks and adding synthetic flavor compounds like cinnamaldehyde to mimic authentic cinnamon flavor [13,14]. More alarmingly, recent investigations by the U.S. Food and Drug Administration have uncovered intentional adulteration of ground cinnamon with lead chromate, a toxic colorant added to enhance appearance, leading to multiple public health alerts and product recalls [15,16,17].

Various analytical techniques have been established to help detect spice adulteration and verify authenticity. Spectroscopic methods, such as Fourier-transform infrared spectroscopy (FTIR), near-infrared (NIR), and X-ray fluorescence (XRF), enable rapid screening for adulterants by generating characteristic chemical profiles of samples. Chromatographic techniques (GC-MS, LC-MS, HPLC) and DNA barcoding further support the identification and quantification of specific adulterants and plant species, especially when combined with chemometric analysis and microscopy for improved detection accuracy [18,19,20,21,22,23]. In addition to these methods, antioxidant profiling has become increasingly relevant for assessing the quality of spices, as it provides insights into the bioactive compounds present, which can be linked to their geographical origin and production methods, thereby supporting efforts to verify authenticity and traceability [24,25]. However, while effective for detecting adulterants, these approaches often lack the robustness to distinguish the geographical origin and agricultural production method independently.

Consequently, these factors have been reliably evaluated using profiling or fingerprinting strategies, such as stable isotope and elemental analysis, which, when combined with chemometrics, have proven effective for ensuring accurate traceability and verification. The elemental profile of a spice reflects the soil in which it is cultivated. Additionally, stable isotope ratios of light elements (^2^H/^1^H, ^13^C/^12^C, ^15^N/^14^N, ^18^O/^16^O, ^34^S/^32^S) offer information on plant type or diet (carbon and nitrogen isotopes), geographic origin (hydrogen and oxygen isotopes), and production methods (e.g., organic vs. conventional), serving as robust markers for tracing these factors [26,27]. Previous studies have demonstrated the utility of stable isotope ratios, such as *δ*^13^C and *δ*^15^N, for distinguishing geographical origin and cultivation practices, and *δ*^18^O and *δ*^2^H for tracing provenance and water sources in bell pepper (*Capsicum annuum*) [28,29].

Radiogenic isotopes, such as strontium (Sr), also provide valuable information for tracing the geochemical provenance of food products like spices. For example, Brunner et al. [30] demonstrated that combining multi-elemental analysis (including Rb, Sr, Y, Zr, Mo, Cd, Ba, Pb, Th, U, Mg, Ca, Sc, Ti, Cr, Mn, Fe, Co, Ni, Cu, Zn, As, and rare earth elements) with radiogenic strontium isotope ratios (^87^Sr/^86^Sr) creates a unique fingerprint for authentic Szegedi paprika, effectively determining its geographical origin. The study by Brunner et al. remains the primary and most frequently cited example of integrating multi-elemental profiling and stable isotope ratios for the authentication and determination of paprika’s origins. In contrast, most studies on paprika have focused on either multi-elemental or stable isotope analysis in isolation, without combining both approaches within a single investigation.

FTIR further enhances food authentication by providing rapid, non-destructive analysis. By comparing the unique spectral fingerprints of paprika or cinnamon samples with the reference spectra of authentic substances, adulterants such as spent paprika or coffee husks can be detected. FTIR allows for the simultaneous analysis of multiple sample components, offering a comprehensive overview of chemical composition [31]. For instance, Lixourgioti et al. [32] demonstrated the effectiveness of FTIR combined with chemometric classification for authenticating cinnamon and detecting adulteration in the cinnamon supply chain.

Building on the strengths of individual analytical techniques, this study introduces an integrated authentication strategy that combines stable isotope analysis, multi-elemental profiling, FTIR fingerprinting, and complementary antioxidant profiling. This comprehensive approach enables the robust detection of adulteration and precise verification of geographical origin and production methods for paprika and cinnamon. Notably, this is the first application of stable isotope-based authentication to cinnamon. By applying this framework to Slovenian market samples, we aim to establish a reliable, multidimensional model to support future spice authentication and traceability initiatives.

## 2. Materials and Methods

### 2.1. Sample Collection

Ninety-one spice samples, consisting of forty-five paprika and forty-six cinnamon samples, were purchased from Slovenian markets and online stores. The selection was diverse regarding type, texture, geographical origin, and agricultural methods, as detailed in the Supplementary Material (Tables S1 and S2).

The paprika samples included sweet, hot, and smoked varieties, with some lacking specific variety identification. Among the 45 samples, 17 were sweet, 12 were hot, 11 were smoked (3 were sweet and 3 were hot), and 5 were of unidentified varieties. The geographical distribution of the samples was as follows: Hungary (n = 5), Serbia (n = 4), Spain (n = 22), Peru (n = 2), India (n = 1), and China (n = 2). Five samples had no origin information, while three were blends from multiple origins or labeled as EU agriculture. Five samples were labeled as organic.

Cinnamon samples varied in geographical origin, agricultural method, price, packaging weight, and type of packaging. Nineteen were labeled as organic. The origin distribution included Sri Lanka (13), Madagascar (5), Indonesia (8), Vietnam (2), China (1), and India (1). Seven samples had no specified geographical origin, and nine were labeled non-EU agricultural products or mixtures of different origins. Samples included 38 ground cinnamon samples and 8 cinnamon sticks (whole bark). Twenty-three were identified as Ceylon cinnamon and five as cassia.

### 2.2. Preparation of Extracts

Known weights (5 g) of paprika powder and ground cinnamon sticks were wrapped in cling film and frozen at −80 °C for 1 h. The frozen samples were then lyophilized. For extraction, 3.00 ± 0.01 g of the lyophilized sample was mixed with 15 mL of 70% ethanol using a vortex. The mixture was placed on a shaker for 2 h, with vortex mixing every 30 min. It was then sonicated in an ultrasonic bath for 30 min, with intermittent vortex mixing, followed by an additional 3 h of shaking with vortex mixing every 20 min. Once extracted, the mixture was filtered (Sartorius, Göttingen, Germany) into a 15 mL centrifuge tube covered in aluminum foil. The extracts were centrifuged at 4000 rpm for 10 min, and the supernatant was stored at 4 °C. Before analysis, the extracts were brought to room temperature (25 °C) and centrifuged for 5 min at 4000 rpm.

### 2.3. DPPH Radical Scavenging Activity Analysis

Antioxidant activity was determined using the DPPH radical scavenging method [33]. In brief, 0.1 mL of extract was diluted with 70% ethanol (15 mL) and added to 2.9 mL of a DPPH (diluted 50-fold for the assay) in ethanol. The DPPH solution was prepared on an ongoing basis, with a freshly prepared working solution to ensure the stability and reliability of the radical concentration, as the DPPH radical concentration decreases over time, even when stored protected from light and in a refrigerator. The mixture was vortexed and incubated in the dark for 30 min at room temperature (25 °C). A control sample (0.1 mL of 70% ethanol + 2.9 mL DPPH solution) was prepared similarly. After incubation, absorbance was measured at 517 nm using a UV-Vis spectrophotometer (Hewlett-Packard, Palo Alto, CA, USA). Color interference was assessed by performing background absorbance (517 nm) measurements of the dark-colored samples. Dark-colored spice matrices can introduce significant matrix interferences in spectrophotometric assays like the DPPH radical scavenging method. These interferences arise from the intrinsic coloration and polyphenolic content of the sample itself, which may absorb at or near the same wavelength used to measure DPPH activity. As a result, the absorbance attributable to DPPH reduction can be confounded by background absorbance from the extract, leading to over- or underestimation of antioxidant activity. To mitigate this, we included sample blanks to correct for background absorbance and used diluted samples to minimize color intensity without exceeding assay linearity. Antioxidant activity was expressed as trolox equivalents (mg trolox per gram of dry sample).

### 2.4. Total Phenolic Content Analysis

The total phenolic content was determined using the Folin–Ciocalteu method [34] (Gutfinger, 1981). A 0.2 mL aliquot of the extract, diluted in 70% ethanol (15 mL), was mixed with 0.125 mL of Folin–Ciocalteu reagent and left to stand for 5 min. Then, 0.125 mL of 20% sodium carbonate solution was added, and the volume was adjusted to 1 mL with Milli-Q water. The mixture was vortexed, incubated in the dark at room temperature (25 °C) for 40 min, and centrifuged at 13,000 rpm for 10 min. Absorbance was measured at 765 nm using a UV-Vis spectrophotometer (Hewlett-Packard, Palo Alto, CA, USA). Results were expressed as milligrams of gallic acid equivalents per gram of dry lyophilized sample (mg GA/g LV).

### 2.5. Fourier-Transform Infrared Spectroscopy (FTIR)

The IR spectra were obtained using Fourier transform infrared spectroscopy (FTIR) with an ATR-FTIR Frontier spectrometer (Perkin Elmer, Shelton, CT, USA). No prior sample preparation was required except for grinding non-powdered paprika and cinnamon samples. All samples were stored in a dry, dark place until analysis. Samples were pressed ensuring the pressure of 60 kp for samples of paprika and 70 kp for cinnamon on the ATR measurement cell to ensure optimal contact with the diamond crystal. Spectra were recorded at room temperature (25 °C) in the mid-infrared range of 4000 to 450 cm^−1^ with a resolution of 4 cm^−1^. Each spectrum was averaged over 32 scans to improve the signal-to-noise ratio [35]. Prior to each measurement, a background spectrum was recorded using a clean, dry ATR crystal and automatically subtracted using the instrument’s Spectrum IR software (Spectrum IR, version 10.6.2.1159, Perkin Elmer). After each scan, the sample was removed, and the cell was cleaned with wipes soaked in propan-2-ol to prevent cross-contamination. The final IR spectra were recorded and analyzed using Spectrum IR software (Spectrum IR, version 10.6.2.1159, Perkin Elmer) to identify signal differences between samples.

### 2.6. Inductively Coupled Plasma Mass Spectrometry (ICP-MS)

Elemental concentrations were determined using an inductively coupled plasma mass spectrometer (ICP-MS) (Agilent 8800, Santa Clara, CA, USA), following the optimized method described by Potočnik et al. [36]. Samples were first decomposed in an UltraWAVE microwave decomposition system (MILESTONE Srl, Sorisole Italy), which allows for high-pressure, high-temperature digestion in a closed-vessel environment to ensure complete sample dissolution and minimize contamination. A known amount (0.08 g) of sample and 1 mL of 65% HNO_3_ were added to a clean Teflon digestion vessel. The decomposition was performed as follows: samples were first heated to 240 °C for 20 min, maintained at 240 °C for 15 min and then allowed to cool to 40 °C. The maximum pressure was set to 100 bars. Nitrogen (N_2_) was used as the loading gas at room temperature (25 °C) and pressure (25 bar). The decomposed samples were transferred into plastic vials and made up to 10 mL with Milli-Q water. In the case of the sediment at the bottom of the vial, the sample was filtered through a hydrophilic 0.45 μm filter (Sartorius, Germany) and diluted (1:5 and 1:10) with 5% HNO_3_.

Two reference materials were also analyzed: tomato (SRM 1573a), peach (SRM 1547), and spinach leaves (SRM 1570a). The detection limits for the selected elements are as follows: Na (2 µg/g), Mg (0.5 µg/g), Al (5 µg/g), P (0.4 µg/g), S (2.2 µg /g), K (11 µg/g), Ca (22,000 ng/g), V (1 ng/g), Cr (9.5 ng/g), Mn (17 ng/g), Fe (600 ng /g), Co (1 ng/g), Ni (35 ng/g), Cu (25 ng/g), Zn (450 ng/g), As (3 ng/g), Se (5 ng/g), Rb (6 ng/g), Sr (130 ng/g), Mo (5 ng/g), Ag (2 ng/g), Cd (1.5 ng/g), Cs (0.6 ng /g), Ba (50 ng/g), Hg (0.7 ng/g), and Pb (15 ng/g). Replicate measurements showed good reproducibility, with relative standard deviations (RSD) generally below 5%, confirming the reliability of the ICP-MS data. An RSD of 5–10% was considered acceptable for elements present at concentrations near the detection limit.

### 2.7. Isotope Ratio Mass Spectrometry (IRMS)

The isotopic ratios of ^13^C/^12^C, ^15^N/^14^N and ^34^S/^32^S values were determined using isotope ratio mass spectrometry (IRMS). The results were expressed as *δ*^13^C, *δ*^15^N and *δ*^34^S. Measurements were made using a Vario IsoPrime100—Vario PYRO Cube (OH/CNS Pyrolyser/Elemental Analyzer; IsoPrime, Cheadle, Hulme, UK)). Samples were prepared by weighing a known amount of lyophilized sample (5 mg) into a tin capsule together with an equal amount of tungsten (VI) oxide (WO_3_) as a combustion accelerator. The tin capsule was then sealed and placed into the elemental analyzer. International and laboratory reference materials with a known isotopic composition were used for quality control.

The results for carbon were normalized according to the following international reference substances: USGS91 (rice flour with a weight of 6.0 mg) with a value *δ*^13^C of −28.28 ± 0.08‰ and USGS89 (porcine collagen with a weight of 6.0 mg) with a value *δ*^13^C of −18.13 ± 0.11‰. Samples were normalized for nitrogen according to the following international reference materials: USGS91 with a value *δ*^15^N of 1.78 ± 0.12‰, USGS43 (Indian human hair powder with a weight of 1.0 mg) with a value *δ*^15^N of 8.44 ± 0.10‰ in USGS61 (1.0 mg weighed caffeine) with a *δ*^15^N value of −2.87 ± 0.04‰. The laboratory reference material CRP-IAEA (casein protein, 2.8 mg) with a *δ*^13^C value of −20.34 ± 0.09‰ and a *δ*^15^N value of 5.62 ± 0.19‰ was used for the control. In the case of sulfur, the reference materials USGS43, with a *δ*^34^S value of 10.46 ± 0.22‰, and USGS91, with a *δ*^34^S value of −20.85 ± 0.72‰, were used to normalize the results. The control was the CRP-IAEA laboratory reference material with a *δ*^34^S value of 4.18 ± 0.79‰. The measurement error for determining *δ*^13^C and *δ*^15^N values was ± 0.2‰ and ± 0.3‰ for *δ*^34^S.

### 2.8. Statistical Data Processing

All elemental and *δ*^13^C, *δ*^15^N and *δ*^34^S data were statistically analyzed using Microsoft Excel, XLSTAT software package (ADDinsoft, New York, USA, 2019) and SIMCA-P (Soft Independent Modeling by Class Analogy; SIMCA 17.0.2, Sartorius Stedim Biotech, Umeå, Sweden) to identify differences between datasets. In XLSTAT, the Kruskal–Wallis test was performed for multiple groups based on geographical origin, and the Mann–Whitney test was applied for two groups based on the agricultural production method since the data did not follow a normal distribution. Additionally, discriminant analysis (DA) was used to enhance group differentiation. In SIMCA, Orthogonal Partial Least Squares Discriminant Analysis (OPLS-DA) was used to reveal patterns and relationships within the data. Candidates for discriminant markers were chosen according to the Variable Importance in Projection (VIP) values derived from the OPLS-DA models. VIP values > 1 were significant and served as the cutoff for identifying the most relevant discriminant markers to differentiate between groups.

## 3. Results and Discussion

### 3.1. Paprika

#### 3.1.1. Antioxidant Activity (AA) and Total Phenolic Compound (TPC) Content

Antioxidant activity values ranged from 2.36 ± 0.08 mg TE/g LV to 4.65 ± 0.07 mg TE/g LV, with the highest values recorded in P1 (4.65 ± 0.07 mg TE/g LV), P15 (4.60 ± 0.07 mg TE/g LV), and P2 (4.52 ± 0.05 mg TE/g LV). These samples represented non-organic sweet paprika from Hungary and Peru, indicating that these growing regions may provide favorable conditions for maintaining a high antioxidant capacity. Conversely, the lowest AA values were observed in P2 (2.87 ± 0.06 mg TE/g LV), P42 (2.43 ± 0.07 mg TE/g LV), and P16 (2.36 ± 0.08 mg TE/g LV), which included hot and sweet paprika samples from Spain, Serbia, and India.

The TPC content in paprika samples ranged from 7.5 ± 0.2 mg GA/g LV to 16.2 ± 0.2 mg GA/g LV, with substantial variation across different samples. The highest values were observed in P27 (16.2 ± 0.2 mg GA/g LV, fragmented paprika from Spain), P25 (15.4 ± 0.1 mg GA/g LV, organic sweet paprika from Spain), and P22 (15.0 ± 0.1 mg GA/g LV, organic smoked paprika from Spain). Notably, P27, which was in fragmented form rather than ground, exhibited the highest retention of phenolic compounds, which may be due to its reduced surface area exposure, minimizing oxidation and degradation. The lowest TPC levels were recorded in P42 (7.5 ± 0.2 mg GA/g LV, sweet paprika from Spain), P16 (8.1 ± 0.1 mg GA/g LV, hot paprika from India), and P39 (8.3 ± 0.2 mg GA/g LV, sweet paprika from Serbia). The antioxidant activity (AA) and total phenolic content (TPC) of ground paprika samples are presented in Table S3 of the Supplementary Material.

Despite the observable differences among the samples, no strong correlation was found between geographical origin, agricultural production method, paprika variety, and phenolic content. Likewise, no statistically significant difference was observed between organic and conventional paprika regarding antioxidant potential, suggesting that the agricultural production method alone does not substantially influence radical scavenging activity. Additionally, hot paprika varieties did not consistently exhibit higher antioxidant activity than sweet varieties, indicating that capsaicinoid content may not be the primary driver of antioxidant potential. Instead, it is possible that post-harvest processing techniques, drying conditions, and storage duration play a more dominant role in polyphenol retention and antioxidant activity, as these factors are known to influence polyphenol stability. These findings align with previous studies [6,37,38,39], which highlight the significant influence of environmental and processing factors on the bioactive compound profile of paprika.

#### 3.1.2. Correlation Between Antioxidant Activity and Total Phenolic Content

A moderate correlation (R^2^ = 0.555) was found in paprika samples, indicating that a higher TPC generally corresponded to higher AA. However, TPC values often exceeded AA values, suggesting that the Folin–Ciocalteu method might overestimate phenolic content due to interactions with non-phenolic compounds such as sugars and amino acids. Additionally, other compounds in paprika, including carotenoids, vitamins C and E, and capsaicinoids, may significantly contribute to antioxidant activity, influencing the observed correlation.

#### 3.1.3. FTIR Results

**Figure 1:**
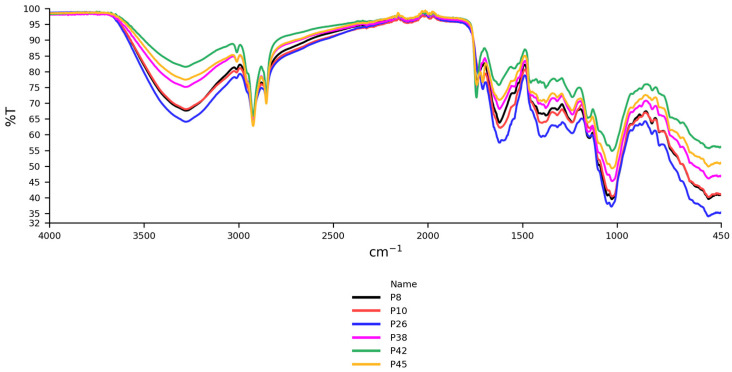
IR spectra of sweet pepper samples of Spanish origin (P8, P10, P26, P38, P42, P45).

Highly similar IR spectra were observed across all analyzed ground paprika samples, with some minor differences. For representative presentation, the IR spectra of non-organic sweet peppers from Spain (P8, P10, P38, P42, P45), along with crushed sweet peppers (P26) of the same origin and conventional production, are illustrated in Figure 1.

The spectral analysis identified key peaks in both the functional group (3600–1200 cm^−1^) and fingerprint (1200–600 cm^−1^) regions, detailed in Table S5 of the Supplementary Material. Notable peaks were observed at 3280, 2920, 1740, 1622, and 1030 cm^−1^, with the most prominent at 1027 cm^−1^. Minor peaks appeared at 1236, 1147, and 816 cm^−1^ [40,41].

A broad band (3600–3000 cm^−1^) with a peak at 3280 cm^−1^ was linked to O–H stretching vibrations, likely from water or polyphenols [41,42]. A smaller band at 3010 cm^−1^ was only notable in samples P19, P41, P42, P44, and P45. Peaks at 2950–2800 cm^−1^ were tied to C-H stretching in CH_3_ and CH_2_ groups. Peaks at 2920 and 2850 cm^−1^ were assigned to CH_2_ stretching vibrations [40], while a peak at 2110 cm^−1^ (C≡C stretching) may result from crystal-enhanced ATR-FTIR. Additionally, a weak band was observed above 3000 cm^−1^, which could be attributed to aromatic C-H stretching or possibly to non-conjugated achene double bonds.

The 1743 cm^−1^ peak was attributed to C=O stretching vibration, might be associated with oil resin components like capsanthin and capsorubin [43]. Its presence varied, with it being absent in several samples (e.g., P10, P11, P14), indicating possible adulteration. Samples that lacked this peak also exhibited a peak shift to 1710 cm^−1^, which may correspond to capsaicin. These differences support previous findings linking genuine paprika to carotenoid peaks, which are often missing in altered samples [44].

**Figure 2:**
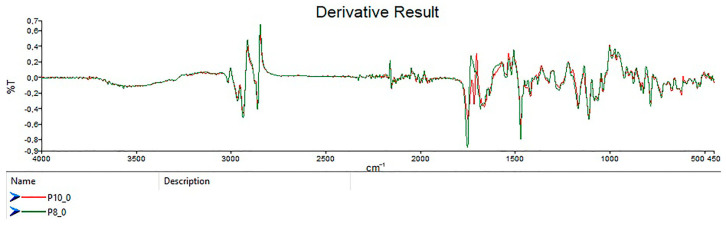
Derivatives of the IR spectra for sweet ground paprika samples from Spain P8 and P10.

**Figure 3:**
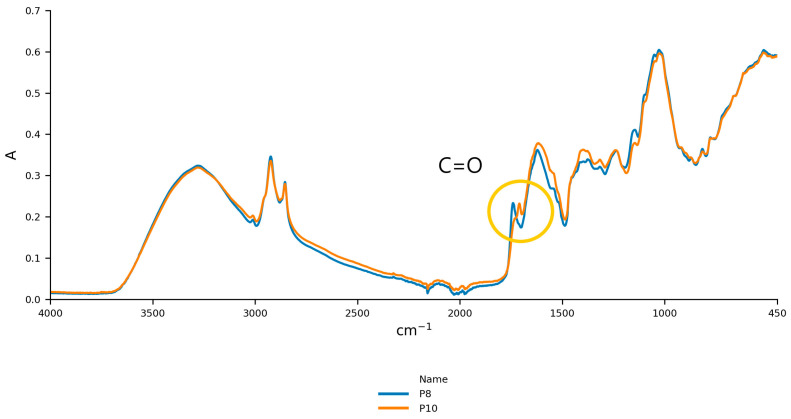
The peaks at 1743 cm^−1^ observed in our paprika samples (P8, P10) are similar to those reported in ground paprika by Galvin-King et al. [44], attributed to C=O stretching vibrations, as marked by the yellow circle.

The first derivative spectra for P8 and P10 (Figure 2) and comparisons with Galvin-King et al. [44] (Figure 3) show notable spectral differences, reinforcing variations in counterfeit samples.

In the fingerprint region, the dominant peak at 1027 cm^−1^ was consistent across samples, related to C-C vibrations and carbohydrate-related oscillations [37]. Peaks at 1236 and 1147 cm^−1^ were also found but were less pronounced in some samples (e.g., P10, P11). A minor peak at 816 cm^−1^, likely from C-H or O-H deformations, was found in several samples [40].

In samples lacking the 1743 cm^−1^ peak, peaks at 1377 and 1400 cm^−1^ were observed, showing compositional differences. The 1377 cm^−1^ peak (symmetrical CH_3_ deformation vibrations) was absent or weak in several samples, while the 1400 cm^−1^ peak, hypothetically linked to azo dyes like solar yellow, E110, was observed in these samples [40]. Azo dyes, though allowed in some foods, are banned in ground paprika due to their potential to mask quality issues.

Most samples with a 1400 cm^−1^ peak also lacked the 1743 cm^−1^ peak, suggesting synthetic dye use to replace natural pigments. The 1400 cm^−1^ peak may also signal use of leftover peppers post-resin extraction. For instance, P26 (crushed, not powdered) showed a strong 1400 cm^−1^ and weak 1377 cm^−1^ peak, possibly reflecting processing or storage effects. These hypotheses require further chemical confirmation.

A similarity analysis of paprika samples focusing on origin, variety, and production method (Table S6; Supplementary Materials) found strong correlations (0.996) in some pairs (e.g., P38–P6, P15–P35) and weaker ones (0.706) in others (e.g., P42–P26). Samples without the 1743 cm^−1^ peak showed high mutual similarity (0.920–0.991), while samples with this peak also clustered tightly (0.902–0.996), suggesting its critical role in sample grouping.

FTIR analysis showed that smoking, origin, and production method had little effect on overall composition. Differences in FTIR were minor, similarly to AA and TPC. The variability of paprika samples from different producers was comparable to that observed within samples from a single producer, suggesting that production processes and origin contribute minimally to the differences in paprika composition, aligning with Horn et al. [43].

#### 3.1.4. Multi-Elemental Composition

The elemental composition of 45 ground paprika samples was categorized (Table 1) into macroelements (Na, Mg, Ca, P, S, K), microelements (Fe, Zn, Cu, Mn), and toxic elements (Hg, Cd, Pb, As).

In the paprika samples, the average elemental distribution (mg/kg) was K (26,707) > P (3795) > Mg (2571) > S (2229) > Ca (2204) > Na (414) > Fe (258) > Al (238) > Zn (20.8) > Mn (19.1) > Sr (17.4) > Rb (16.5) > Cu (11.0) > Ba (3.56) > Cr (1.91) > Ni (1.39) > V (0.45) > Co (0.29) > Pb (0.19) > As (0.11) > Cd (0.096) > Cs (0.071) > Se (0.065) > Ag (0.013) > Hg (0.0017). Similar patterns were found in previous studies on ground paprika [45].

The elemental composition of ground paprika revealed that Cr levels ranged from 0.16 to 5.74 mg/kg, exceeding the range reported by Garcia et al. [46] (0.19–0.65 mg/kg). Similarly, Al content was significantly higher (18–629 mg/kg) compared to the values reported by Lopez et al. [47] (12–43.6 mg/kg). Sweet peppers demonstrated higher levels of Fe (75.9–534 mg/kg; mean 272 mg/kg) than hot peppers (96.7–607 mg/kg; mean 232 mg/kg), aligning with findings by Palacios-Morillo et al. [45]. Potassium was the most abundant element, with average concentrations in sweet peppers (27.5 g/kg) slightly higher than in hot peppers (25.7 g/kg), consistent with Hungarian paprika samples studied by Ördög et al. [48]. While Mn and Mg content showed minimal variation between sweet and hot peppers, our results indicated a higher Mo concentration in sweet peppers (448 μg/kg) compared to hot peppers (323 μg/kg), differing from the results found by Ördög et al. [48].

Regarding toxic elements, lead (Pb) concentrations in ground paprika were below the EU limit of 0.60 mg/kg (Commission Regulation (EU) 2021/1317), with the highest concentration (0.537 mg/kg) in P26 (crushed sweet pepper from Spain) and the lowest (<0.015 mg/kg) in P23 (organic sweet pepper from Spain). Cadmium (Cd) levels ranged from 0.028 to 0.318 mg/kg, with some samples exceeding regulatory thresholds after applying conversion factors for dry weight. Arsenic was highest in P19 (0.183 mg/kg), while Hg content was negligible, with the maximum detected at 0.003 mg/kg in P24.

#### 3.1.5. Stable Isotope Composition of Light Elements

The measured *δ*^13^C values in paprika samples ranged from −29.7 ± 0.1‰ to −26.4 ± 0.1‰, consistent with the typical isotopic signature of C3 plants. All other samples fell within this range. The highest *δ*^15^N value was found in sample P23 (11.1 ± 0.04‰), while the lowest was in sample P29 (1.8 ± 0.2‰). The *δ*^34^S values varied between 2.3 ± 0.2‰ and 11.1 ± 0.2‰, considering paprika from various geographical origins and agricultural practices. Compared with previous studies, Mahne Opatić et al. [49] reported a *δ*^34^S range of 1.3‰ to 15.0‰ for sweet peppers from multiple countries, aligning with the present study findings (*δ*^34^S range: 3.0–11.1‰). Their reported averages for *δ*^34^S were Slovenian (2.9‰), Spanish (5.8‰), Italian (6.4‰), Moroccan (8.4‰), and Greek (12.2‰). The *δ*^13^C values for the same countries were: −27.5‰ (Slovenia), −27.7‰ (Spain), −27.4‰ (Italy), −26.9‰ (Morocco), and −25.8‰ (Greece). For *δ*^15^N: Slovenian (6.0‰), Spanish (3.1‰), Italian (−4.1‰), Moroccan (1.9‰), and Greek (2.4‰). Spanish paprika, including both organic and non-organic samples, produced comparable averages: *δ*^13^C (−28.1 ± 0.03‰), *δ*^15^N (4.8 ± 0.1‰), and *δ*^34^S (8.3 ± 0.1‰).

#### 3.1.6. Differentiating Samples According to the Country of Origin

These results supported the use of multivariate approaches for origin discrimination. However, due to limited sample availability, China and Peru were excluded from further modeling. Discriminant analysis (DA) was subsequently used to assess the geographical origin of paprika samples from Hungary, Serbia, and Spain, based on isotopic ratios (*δ*^13^C, *δ*^15^N, *δ*^34^S) and elemental composition.

**Figure 4:**
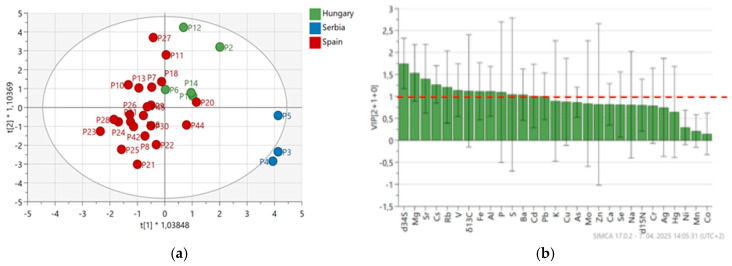
OPLS-DA models differentiating geographical origins (Hungary, Serbia, Spain) based on isotopic ratios and elemental composition: (**a**) score plot illustrate sample clustering by country—Hungary (green), Serbia (blue), and Spain (red)—with a 95% confidence interval ellipse; (**b**) Variable Importance in Projection (VIP) plot highlight the most influential variables (VIP > 1, red dashed line) for group discrimination.

Thirty samples were analyzed, including sweet and spicy varieties produced via organic and conventional methods. Spanish samples formed the most distinct and well-separated group, followed by Serbian samples. In contrast, Hungarian samples exhibited some overlap with the Spanish group, with their centroids positioned close together (Figure 4). Twelve isotopic and elemental variables—*δ*^34^S, Mg, Sr, Cs, Rb, V, *δ*^13^C, Fe, Al, P, S, and Ba—were identified as the most influential for origin classification, as shown by variables with VIP scores > 1 (Figure 4). These results align with earlier studies, including those of Brunner et al. [30] and Fiamegos et al. [50,51], who also demonstrated the effectiveness of these markers in paprika provenance analysis. The obtained OPLS-DA resulted in two predictive and one orthogonal component (2 + 1), producing an R^2^X = 0.476, R^2^Y = 0.567, and Q^2^ = 0.226. Classification metrics (sensitivity, specificity, precision, F1 score) were calculated for each class individually and summarized using macro average. The F1 score rate, obtained by internal cross-validation, was 83.6%, sensitivity was 80.0%, specificity was 87.5%, precision was 96.0%, and accuracy was 90.0%.

Spanish samples were characterized by distinctively higher *δ*^13^C and Sr values, suggesting differences in climate (e.g., aridity and sunlight intensity) and soil mineral composition, likely reflecting the more calcareous or marine-influenced soils found in parts of Spain. Higher *δ*^34^S values may also indicate the use of sulfur-rich fertilizers or the influence of proximity to marine environments. Serbian samples exhibited the highest *δ*^15^N values (5.2‰) and S content (2465 mg/kg), likely due to fertilizer use, while Hungarian samples displayed intermediate values for most variables. Elements such as Mg, Fe, Al, and P—which are present to some extent in samples from both Hungary and Spain due to similar soil types and farming techniques [48]—still contributed to the separation when evaluated in combination with other variables. This phenomenon is supported by studies showing that agricultural practices and soil management can have a greater impact on the elemental composition of crops than geographic proximity alone [30,50]. Trace elements like Cs, Rb, and Ba, which are often influenced by the underlying geology and long-term land use, provided further resolution. Despite some misclassification among Hungarian samples, the model exhibited strong discriminatory power, with statistically significant separation by origin. Serbian and Spanish samples were classified with perfect accuracy (100%), whereas Hungarian samples showed lower accuracy, with only 40% correctly classified.

Closer inspection of the score plot revealed that several Spanish samples (P11, P18, P20, and P27) clustered near the Hungarian group. While P20 appeared typical, samples P11, P18, and P27 displayed unusual chemical and spectral characteristics. FTIR analysis showed that these samples lacked the characteristic 1743 cm^−1^ absorption band associated with paprika oleoresins, suggesting the presence of potential residual plant matter or unauthorized coloring agents. These anomalies may indicate low-quality or adulterated material, which could distort the chemical profiles and obscure true geographic signals.

In contrast, Serbia’s more isolated agricultural context likely preserved a distinct chemical fingerprint, supporting its excellent classification. Lastly, since all paprika samples were commercially sourced without certified origin documentation, the possibility of mislabeling or origin fraud cannot be excluded and should be considered when interpreting these findings. Further, model evaluation was conducted with full consideration of the sample size imbalance, and the results were interpreted cautiously. This limitation highlights the need for more balanced and representative sampling in future studies to ensure greater reliability and generalizability of the classification models.

#### 3.1.7. Differentiating Samples According to the Agricultural Production Practice

A Mann–Whitney test confirmed statistically significant differences (*p* < 0.05) in elemental composition and isotope ratios between organic (n = 5) and conventional (n = 37) paprika samples. Conventional samples exhibited higher concentrations of Na, Al, V, Cr, Fe, Rb, Cs, Ba, and Pb, likely due to metal-based fertilizers, pesticides, and environmental contamination. In contrast, organic samples were characterized by elevated Zn, *δ*^15^N, and *δ*^34^S values, reflecting the use of organic fertilizers.

**Figure 5:**
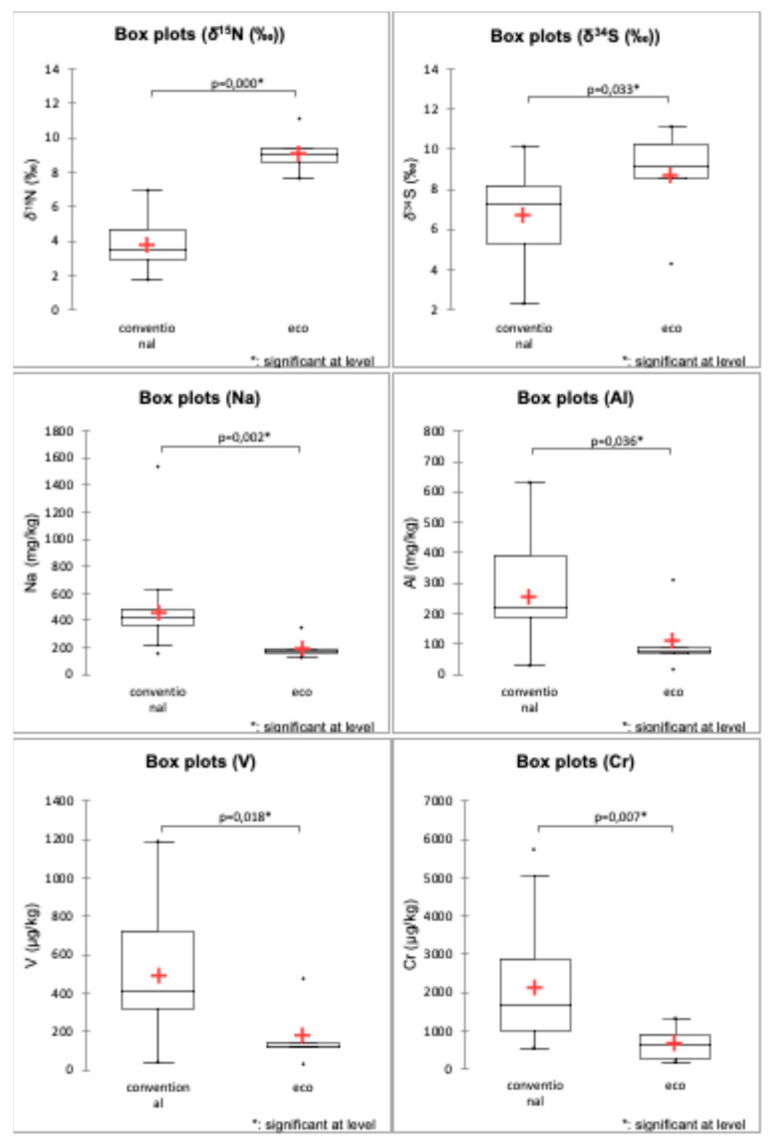
Element concentrations (Na, Al, V, Cr), *δ*^15^N, and *δ*^34^S values in paprika samples according to the agricultural production method, where statistical differences were observed. The red “+” symbol indicates the mean value for each group.

Toxic element levels, particularly As and Pb, were significantly higher in conventional samples, with Pb concentrations averaging 213 μg/kg in conventional samples compared to 79 μg/kg in organic samples. Box plots of concentrations of elements (Na, Al, V, Cr) and values *δ*^15^N and *δ*^34^S in samples can be seen in Figure 5, while box plots of other elements—Fe, Zn, As, Rb, Ba, Pb, and Cs—can be found in the Supplementary Materials (Figure S1).

These findings align with those of Fiamegos et al. [50], who observed similar trends in paprika using ED-XRF. Their study found organic samples with higher Mg, K, S, P, Cl, Zn, and Br concentrations, while conventional samples contained higher Fe, Mn, Cr, Ba, and Rb levels. The isotopic marker *δ*^15^N emerged as a reliable indicator of organic farming, with all organic paprika samples exceeding 7‰, consistent with findings by Schmidt et al. and Flores et al. [52,53]. These studies demonstrated that organic fertilizers enriched in ^15^N have higher *δ*^15^N values than synthetic fertilizers, which are nearer to zero. In contrast, *δ*^13^C showed limited utility for distinguishing farming practices, as differences were statistically insignificant.

Perez-Lopez et al. [54] show how agricultural practices significantly influenced the mineral concentrations of paprika grown in greenhouses under controlled conditions, thereby eliminating climatic variability as a factor. They observed higher concentrations of most minerals in organic paprika compared to integrated and conventional products. While their findings partially align with this study, there are notable differences. Their study showed higher Fe concentrations in conventional paprika and no statistically significant differences in Cu or Ca levels between organic and conventional samples. These discrepancies may stem from differences in experimental conditions, such as the greenhouse-controlled environments used in their study versus the commercially sourced samples of various origins in this study, as well as variations in fertilization practices or regional soil compositions.

### 3.2. Cinnamon

#### 3.2.1. Antioxidant Activity (AA) and Total Phenolic Compound (TPC) Content

Cinnamon samples exhibited significantly higher antioxidant activity than paprika samples, with AA values ranging from 53 ± 1 mg TE/g LV to 404 ± 1 mg TE/g LV. The highest AA values were recorded in C24 (404 ± 1 mg TE/g LV, organic ground cinnamon of unknown geographical origin and C21 (341 ± 1 mg TE/g LV, ground cassia cinnamon from Indonesia). In contrast, the lowest AA values were found in C5 (55 ± 1 mg TE/g LV), C23 (53 ± 1 mg TE/g LV), and C11 (56 ± 1 mg TE/g LV), all of which were organic Ceylon cinnamon samples from Sri Lanka.

Cinnamon samples contained significantly higher phenolic content than paprika samples, with TPC values ranging from 33 ± 1 mg GA/g LV to 254 ± 1 mg GA/g LV. The highest levels were found in C24 (254 ± 1 mg GA/g LV, organic ground cinnamon) and C21 (247 ± 1 mg GA/g LV, cassia cinnamon from Indonesia), while the lowest were recorded in C5 (33 ± 1 mg GA/g LV, organic Ceylon cinnamon from Sri Lanka), C23 (40 ± 1 mg GA/g LV, organic Ceylon cinnamon from Sri Lanka), and C11 (40 ± 0.2 mg GA/g LV, Ceylon cinnamon from Sri Lanka). The antioxidant activity (AA) and total phenolic content (TPC) of cinnamon samples are presented in Table S4 of the Supplementary Material.

The botanical species of cinnamon significantly influenced antioxidant activity (AA) and phenolic content, with cassia cinnamon consistently exhibiting higher values than Ceylon cinnamon. The highest AA levels were observed in cassia samples from Indonesia and Vietnam (C21, C12, C33), whereas the lowest were found in Ceylon cinnamon from Sri Lanka and Madagascar. Similarly, cassia cinnamon contained a greater number of phenolic compounds compared to Ceylon cinnamon. It should be noted that the DPPH assay is not specific to phenolic compounds, and the observed antioxidant activity may also reflect the presence of non-phenolic reducing substances such as ascorbic acid or Maillard reaction products. In the case of cassia cinnamon, the high antioxidant activity values may therefore not be solely attributed to phenolic content. Within the Ceylon group, samples from Madagascar demonstrated higher total phenolic content (TPC) than those from Sri Lanka, suggesting that environmental conditions and genetic variation among cultivars play a role in polyphenol composition.

Form of storage also proved critical; cinnamon sticks displayed higher antioxidant activity and phenolic content than ground cinnamon of the same botanical species and origin, likely due to reduced oxidation and better preservation of bioactive compounds during storage. Furthermore, cinnamon from conventional farming exhibited higher AA and TPC values than organic samples, particularly cassia cinnamon, where non-organic samples showed a markedly increased radical scavenging capacity. These trends are consistent with the findings of Lv et al. [42], who attributed the higher antioxidant potential and polyphenol levels in conventional cinnamon to differences in the concentration of free and bound soluble phenolic compounds.

#### 3.2.2. Correlation Between Antioxidant Activity and Total Phenolic Content

Cinnamon samples exhibited a strong correlation (R^2^ = 0.875) between AA and TPC, confirming that phenolic compounds are major contributors to cinnamon’s antioxidant capacity. Most cinnamon samples showed higher AA values than TPC, except for C12 and C10. The strong relationship suggests that the phenolic compounds measured by the Folin–Ciocalteu method play a crucial role in cinnamon’s antioxidant potential. However, bioactive compounds, like proanthocyanidins and trans-cinnamaldehyde, indicate that not all antioxidant activity is derived solely from TPC. Similar findings were reported by Lv et al. [42], who found an even stronger correlation (R^2^ = 0.979) between TPC and AA in cinnamon.

#### 3.2.3. FTIR Results

**Figure 6:**
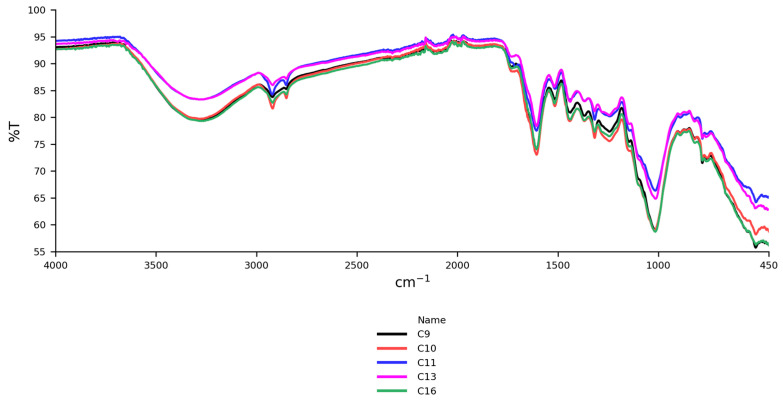
IR spectra of samples of Ceylon cinnamon from Sri Lanka (C9, C10, C11) and Madagascar (C13, C16).

FTIR revealed only minor differences between the cinnamon samples. The IR spectra of non-organic Ceylon ground cinnamon from Sri Lanka (C9, C10, C11) and Madagascar (C13, C16) are shown in Figure 6.

Prominent spectral peaks appeared at 3280, 2920, 1605, and 1015 cm^−1^, with the peak at 1015 cm^−1^ being most intense. Minor peaks were noted at 2110, 1516, 1440, and 780–750 cm^−1^ (Table S7; Supplementary Materials). These indicate chemical composition and functional groups in the samples.

A broad band at 3600–3000 cm^−1^, peaking at 3280 cm^−1^, was linked to O–H stretching vibrations, possibly through water or phenolic compounds like catechin or caffeic acid [35,55]. It could also reflect alcohols (e.g., eugenol) or proteins, as noted by Lixourgioti et al. [32]. This peak was stronger in Ceylon cinnamon, indicating higher phenol and eugenol content, while cassia had an absent or weak peak, suggesting a lower content of phenols and eugenol.

Peaks at 2920 and 2850 cm^−1^ corresponded to asymmetric and symmetric C–H stretching vibrations in CH_2_ groups, likely from lipids or compounds like cinnamaldehyde and eugenol. These were less evident in some samples (e.g., C22, C38), suggesting differences in lipid content or sample preparation.

A strong peak at 1015 cm^−1^, seen across all samples, indicated C–O stretching vibrations in esters and phenolics. The 1605 cm^−1^ peak reflected C=C stretching vibrations in cinnamaldehyde, a major volatile component of cinnamon bark. Some variation in the 1730 cm^−1^ peak (C=O stretching of aldehydes) was observed, with slight shifts in samples (1715 cm^−1^; C10, C11) indicating differences in cinnamaldehyde or other carbonyl compounds. Samples C7 and C24 lacked the peak at 1730 cm^−1^, possibly due to origin or processing method.

#### 3.2.4. Ceylon Cinnamon vs. Cassia

**Figure 7:**
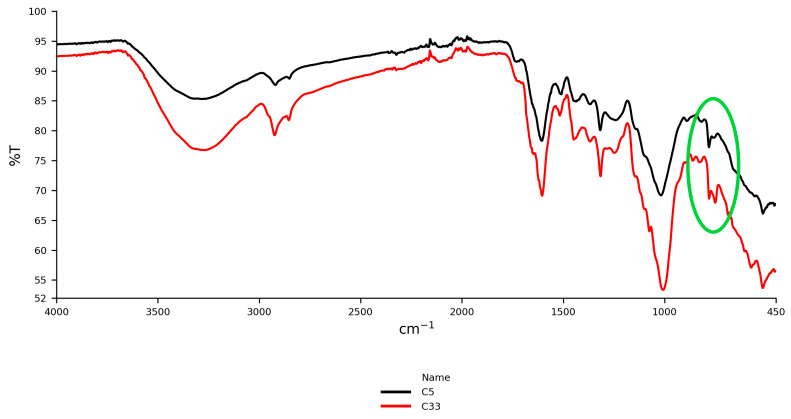
Differentiation of the IR spectrum of cassia (C33) and Ceylon cinnamon from Sri Lanka (C5) at peaks 780 and 750 cm^−1^. The green ellipse emphasizes the spectral region where Ceylon cinnamon exhibits a pronounced peak only at 780 cm^−1^, with no detectable peak at 750 cm^−1^. In contrast, cassia displays distinct peaks at both 750 cm^−1^ and 780 cm^−1^. The absence of a peak at 750 cm^−1^ in Ceylon cinnamon serves as a key spectral marker distinguishing it from cassia.

Peaks between 780 and 750 cm^−1^, representing out-of-plane C–H bending vibrations, were particularly significant for distinguishing between Ceylon cinnamon (*C. verum*) and cassia (*C. cassia*). In Figure 7, the IR spectra of cassia (C33) and Ceylon cinnamon (C5) highlight the differences: cassia samples exhibited a well-defined peak at 750 cm^−1^, while Ceylon cinnamon samples displayed a pronounced peak at 780 cm^−1^. These findings are consistent with previous studies by Li et al. [56] and Yasmin et al. [57], which noted the association of the 750 cm^−1^ peak with coumarin, a compound prevalent in cassia but minimal in Ceylon cinnamon. Thus, this distinct band may serve as a reliable spectral marker for the identification of cassia cinnamon.

#### 3.2.5. Organic vs. Non-Organic Cassia

FTIR revealed notable differences in the fingerprint region between organic and non-organic cassia. Organic samples from Indonesia (C6) and Vietnam (C33) showed distinct peaks at 750 cm^−1^ and 780 cm^−1^, indicating stable phenolic and volatile compound compositions. Non-organic samples from Indonesia (C21, C41) showed a clear peak at 750 cm^−1^ but a weaker one at 779 cm^−1^. Sample C12, also non-organic cassia, had a clear peak at 762 cm^−1^, possibly due to regional variations. Li et al. [56] highlighted that soil, climate, and altitude affect cassia’s essential oil content. Oil cells, the primary sites for synthesizing and accumulating essential oils, tend to exhibit stable compositions, but regional factors can still introduce variability. Still, all non-organic samples of cassia (C12, C21, and C41) shared a weak 779 cm^−1^ peak, setting them apart from organic ones. These findings suggest that organic cassia consistently shows a strong 780 cm^−1^ peak, while non-organic types do not, supporting FTIR’s use in distinguishing them.

#### 3.2.6. Organic vs. Non-Organic Ceylon Cinnamon

**Figure 8:**
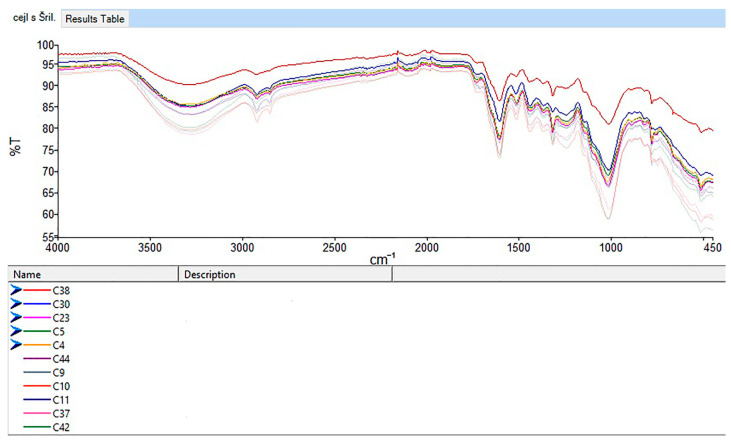
Comparison of IR spectra between organic (C38, C30, C23, C5, C4—indicated by a blue arrow) and non-organic (C44, C9, C10, C11, C37, C42) Ceylon cinnamon samples from Sri Lanka.

Silva Bruni et al. [58] analyzed mid-infrared (MIR) spectra of true (Ceylon) cinnamon and found a key feature for differentiation between organic and non-organic cinnamon samples above 2600 cm^−1^, where O–H stretching and C–H vibrations linked to eugenol and phenolics were evident. Organic cinnamon showed higher transmittance (lower absorbance), indicating lower phenolic content, while non-organic samples showed the opposite (Figure 8). These findings matched this study’s results for cassia and Ceylon cinnamon, especially Sri Lankan samples.

**Figure 9:**
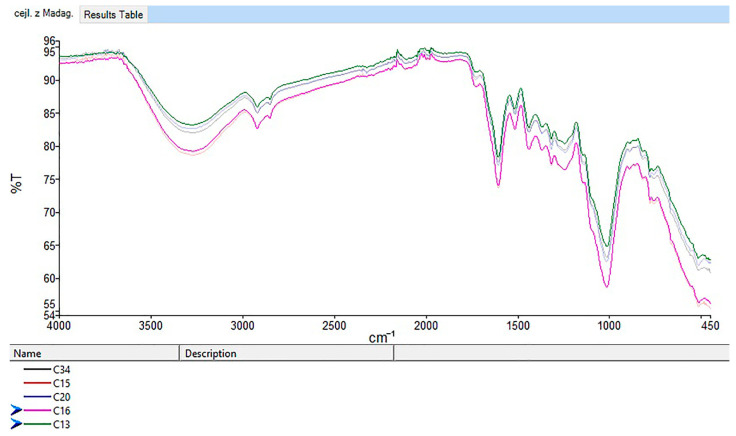
Comparison of IR spectra between organic (C15, C20, C34) and non-organic (C13, C16—indicated by a blue arrow) Ceylon cinnamon samples from Madagascar.

For Sri Lankan Ceylon cinnamon, organic samples showed higher transmittance and thus lower phenolic content, which was confirmed by AA and TPC results. In contrast, Ceylon cinnamon from Madagascar showed inconsistencies: sample C16 (non-organic) resembled organic patterns, while C15 (organic) mirrored non-organic traits (Figure 9). This variation may stem from geography, environment, or processing.

**Figure 10:**
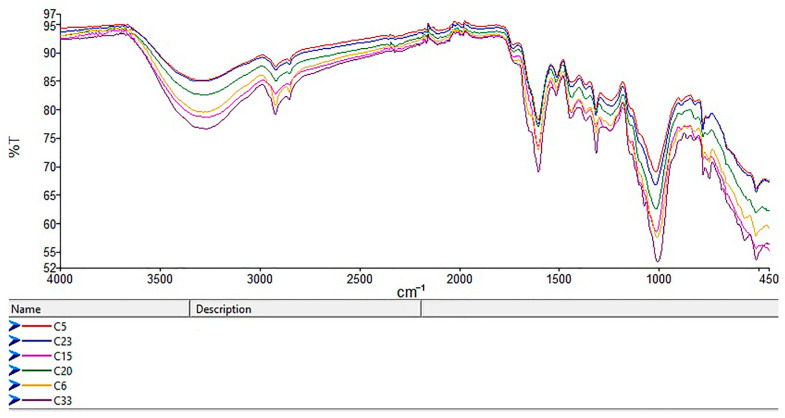
IR spectra of organic samples of cassia (C6, C33) and Ceylon cinnamon from Sri Lanka (C5, C23) and Madagascar (C15, C20).

Cassia samples showed higher AA results and a higher phenolic content than Ceylon cinnamon from Sri Lanka, with lower IR transmittance (e.g., C6, C33 vs. C5, C23). Notably, Madagascar Ceylon samples (C15, C20) had similar transmittance values to cassia, indicating possible compositional overlap (Figure 10).

A similarity analysis among all cinnamon samples with declared species, geographical origin, and production methods showed high correlations (0.94–0.98; Table S8; Supplementary Materials) within the following groups: organic Sri Lanka (C4, C5, C23, C30, C38) and organic Ceylon cinnamon from Madagascar (C15, C20, C34). The strongest correlation was 0.988 (C34 and C20). Non-organic Ceylon samples from Madagascar (C13, C16) also showed strong correlations (0.97–0.98) with other Ceylon cinnamon, despite spectral intensity differences in peaks at 780 and 760 cm^−1^.

Lower values were noted for C37 (0.87–0.93) and C30 (0.91–0.93). Sample C41 showed notably lower similarity (0.76–0.86), indicating significant compositional differences, possibly due to species variation (e.g., *C*. *loureirii* or *C. burmannii*). Cassia samples showed lower correlations with Ceylon cinnamon (0.885–0.928), reflecting species-based differences. High similarity was found within organic cassia from Vietnam (C33) and Indonesia (C6) (0.965) and non-organic cassia from Indonesia (C12, C21) (0.977). C41 again showed lower correlations (0.831–0.848), reinforcing its distinct profile. These findings align with prior research [56], highlighting the influence of both region and species on cassia’s chemical profile.

Overall, FTIR spectra showed strong similarities among cinnamon samples of the same species and production method, indicating stable composition. However, slight differences between organic and non-organic cassia (correlation values: 0.913–0.923) suggest variations in phenolic and volatile compounds. These findings align with AA and TPC analyses, highlighting the influence of species, origin, and agricultural practices on cinnamon’s chemical composition.

#### 3.2.7. Multi-Elemental Composition

The elemental composition of 46 cinnamon samples was categorized (Table 2) into macroelements (Na, Mg, Ca, P, S, K), microelements (Fe, Zn, Cu, Mn), and toxic elements (Hg, Cd, Pb, As).

The average distribution of elements in cinnamon samples, from highest to lowest concentration (mg/kg), was as follows: Ca (12,205) > K (7059) > S (1143) > Mg (833) > P (611) > Al (470) > Na (409) > Fe (331) > Mn (193) > Sr (87) > Ba (80) > Rb (22) > Zn (17) > Cu (6.5) > Cr (1.3) > Ni (0.85) > V (0.67) > Pb (0.61) > Cd (0.24) > Cs (0.23) > Co (0.18) > Mo (0.10) > As (0.042) > Se (0.039) > Hg (0.0095) > Ag (0.0032). These values are consistent with previous research [59,60], which identified calcium as the predominant element in cinnamon.

The elemental composition displayed significant variation. Copper levels ranged from 2.1 to 12.3 mg/kg, lower than the range reported by Goncalves et al. [61] (7.2–21.9 mg/kg). Zinc concentrations (5.4–27.1 mg/kg) were consistent with those observed by Goncalves et al. [61] and Krejpcio et al. [62]. Iron levels (15.4–1680 mg/kg) were substantially lower than those of Singh and Garg [63], who reported 2390 mg/kg for true cinnamon. Similarly, Cr content ranged from 0.1 to 4.7 mg/kg, aligning with the results of Garcia et al. [46] but lower than those obtained by Özcan and Akbulut [60], who detected 7.89 mg/kg in true cinnamon.

Toxic element analysis showed that Pb was highest (4.01 mg/kg) in C25 (ground cinnamon from India), exceeding the EU limit for bark spices (2.00 mg/kg). Cadmium levels ranged from 0.049 to 0.701 mg/kg, with the highest detected levels in C39 (cinnamon sticks of unknown origin). Arsenic ranged from 0.010 to 0.248 mg/kg, while Hg content remained minimal (0.002–0.062 mg/kg). Unlike ground paprika, some cinnamon samples exceeded regulatory Pb limits, suggesting contamination, likely from environmental exposure. Paprika samples displayed higher K and Fe levels, while cinnamon contained higher concentrations of toxic elements, particularly lead and cadmium. This variability is attributed to differences in soil composition, geographic origin, and agricultural practices, as noted in previous studies [61,62,63,64]. Nevertheless, the hazard index (HI), representing the combined non-carcinogenic effects of all toxic elements, did not indicate a potential health risk for cinnamon consumers [65].

#### 3.2.8. Stable Isotope Composition of Light Elements

Cinnamon samples exhibited *δ*^13^C values from −32.6 ± 0.1‰ to −28.9 ± 0.1‰, confirming its classification as a C3 plant. The highest *δ*^15^N value was 7.1 ± 0.1‰, observed in C44, and the lowest was 0.3 ± 0.2‰, found in C9. *δ*^34^S values ranged from −0.9 ± 0.1‰ to 19.1 ± 0.1‰. Although the stable isotope composition of carbon (C), nitrogen (N), and sulfur (S) in cinnamon as a spice has not been previously reported, Sewenig et al. [66] analyzed cinnamon oil and found *δ*^13^C values ranging from −27.1‰ to −25.9‰ for Ceylon cinnamon oils and −26.4‰ to −24.2‰ for cassia oils. Our results support this differentiation.

Organic cassia cinnamon samples had *δ*^13^C values from −29.9‰ to −29.8‰, while conventional samples ranged from −29.6‰ to −29.3‰. The mean *δ*^13^C value for cassia was −29.5‰. Ceylon cinnamon showed greater variation between organic and non-organic samples, ranging from −32.6‰ to −30.1‰. The mean *δ*^13^C value for Ceylon cinnamon was −31.4‰, with samples from Madagascar averaging −32.3‰ and those from Sri Lanka −30.9‰. Samples C37 and C30 were excluded due to discrepancies.

Regarding *δ*^15^N, cassia samples from organic production showed values between 2.0‰ and 4.0‰ (mean 3.0‰), while conventional samples ranged from 1.4‰ to 2.1‰ (mean 1.8‰). The trend was inconsistent for Ceylon cinnamon, with overlapping ranges for organic and conventional samples. Cinnamon *δ*^34^S values also varied significantly. Cassia from organic production ranged from 4.9‰ to 5.1‰ (mean 5.0‰), while conventional samples ranged from 3.9‰ to 5.7‰ (mean 4.8‰). Ceylon cinnamon showed wider variations, particularly in Madagascar, where both organic and conventional samples had high *δ*^34^S values (18.3‰ and 18.1‰, respectively).

#### 3.2.9. Differentiating Samples According to the Country of Origin

**Figure 11:**
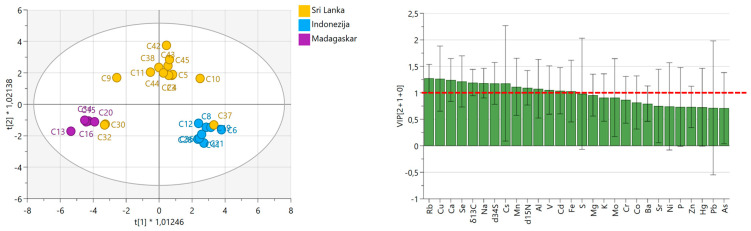
OPLS-DA models differentiating geographical origins (Sri Lanka, Indonesia, Madagascar) based on isotopic ratios and elemental composition. Score plots (**left**) illustrate sample clustering by country: Sri Lanka (yellow), Indonesia (blue), and Madagascar (purple), with the 95% confidence interval ellipse. Variable Importance in Projection (VIP) plots (**right**) highlight the most influential variables (VIP > 1, red dashed line) for group discrimination.

DA was conducted to assess the geographical origin of cinnamon samples from Sri Lanka, Indonesia, and Madagascar, following the exclusion of Vietnam due to limited sample availability. Twenty-seven samples were analyzed, including Ceylon and cassia cinnamon, sourced from conventional and organic production. Indonesian and Madagascan samples formed well-separated groups, while Sri Lankan samples exhibited some overlap, primarily with Madagascan samples (Figure 11). The differentiation of cinnamon samples by origin was primarily driven by fourteen isotopic and elemental variables (Rb, Cu, Ca, Se, *δ*^13^C, Na, *δ*^34^S, Cs, Mn, *δ*^15^N, Al, V, Cd, and Fe) with VIP scores > 1 (Figure 11). These findings partially align with those of Goncalves et al. [61], who identified significant variability in Fe and Al concentrations among cinnamon samples marketed in Portugal. However, their study did not consider geographic or environmental factors, focusing on quality control during production, transport, and storage.

Obtained OPLS-DA comprised two predictive and one orthogonal component (2 + 1), producing an R^2^X = 0.663, R^2^Y = 0.699, and Q^2^ = 0.498. Classification metrics (sensitivity, specificity, precision, F1 score) were calculated for each class individually and summarized using macro average. The F1 score rate, obtained by internal cross-validation, was 88.5%, sensitivity was 92.9%, specificity was 95.7%, precision was 86.8%, and accuracy was 88.9%. Indonesian and Madagascan samples were classified with 100% accuracy, whereas Sri Lankan samples were correctly classified in 78.6% of cases.

Isotopic markers like *δ*^13^C and *δ*^15^N are influenced by climatic conditions and nitrogen sources, respectively, helping to distinguish regions with different rainfall patterns, sunlight exposure, and fertilization methods. *δ*^34^S values can signal the use of sulfate-based fertilizers or proximity to marine environments. For example, Madagascar samples exhibit the highest *δ*^34^S values, probably indicating the influence of the marine environment, while *δ*^15^N values were the highest in Sri Lankan samples, probably influenced by production practices. Elemental markers such as Rb, Cs, and Al are indicative of the local geology, with higher concentrations being observed in the Madagascar sample, while Fe, Cu, and Mn reflect micronutrient availability in soils, which is the lowest in Indonesia. Macronutrients like Ca, Na, and Se also vary depending on soil chemistry, irrigation water composition, and plant uptake efficiency.

Misclassifications involved assigning Sri Lankan samples as Madagascar (e.g., C30) or Indonesia (e.g., C37). FTIR analysis revealed that sample C37, labeled non-organic Ceylon cinnamon from Sri Lanka, deviated markedly from typical Ceylon profiles. It exhibited a weak peak at 780 cm^−1^ and a strong peak at 750 cm^−1^, a pattern characteristic of cassia cinnamon (Figure 8). Antioxidant profiling supported this observation, showing elevated phenolic content consistent with cassia, suggesting possible adulteration or mislabeling.

Sample C30, an organic Sri Lankan cinnamon, displayed weak peaks at 780 cm^−1^ and 750 cm^−1^, resembling the spectral profile of Madagascan samples. This similarity could be attributed to divergent growing conditions, storage or handling issues, analytical variation, or incorrect origin labeling, complicating its classification. These results help explain the observed misclassifications. The cassia-like characteristics of C37 likely led to its misclassification as Indonesian cinnamon, while the overlap between C30 and Madagascan profiles contributed to its incorrect assignment. Environmental factors and the commercial sourcing of uncertified samples may have further contributed to these overlaps.

#### 3.2.10. Differentiating Samples According to the Agricultural Production Practice

**Figure 12:**
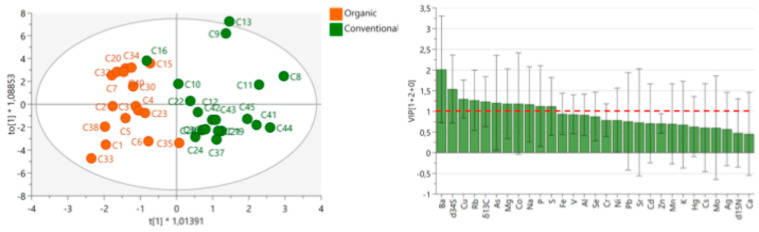
OPLS-DA models showing the differentiation between conventional (green) and organic (eco; orange) cinnamon samples based on isotopic ratios and elemental composition. The 95% confidence interval ellipse is shown. Variable Importance in Projection (VIP) plots (below) highlight the most influential variables (VIP > 1, indicated by the red dashed line) for group discrimination.

OPLS-DA effectively differentiated organic and conventional cinnamon production methods across various origins and species. Figure 12 illustrates the separation, with organic (eco) and conventional samples grouped within a 95% confidence ellipse. Key discriminating variables (VIP > 1), displayed above the red dashed line, include Ba, *δ*^34^S, Cu, Rb, *δ*^13^C, As, Mg, Co, Na, P, and S. Obtained OPLS-DA resulted in one predictive and two orthogonal components (1 + 2), producing an R^2^X = 0.483, R^2^Y = 0.734, and Q^2^ = 0.475. The F1 score rate obtained by internal cross-validation was 94.1%, sensitivity was 94.1%, specificity was 94.7%, precision was 94.1%, and accuracy was 94.4%.

While most samples were correctly classified, discrepancies emerged. Sample C16, labeled as conventional, aligned more closely with organic cinnamon, particularly Ceylon cinnamon from Madagascar (correlation: 0.971), suggesting possible mislabeling or a transition to organic production. FTIR analysis supports this assumption (Figure 9).

**Figure 13:**
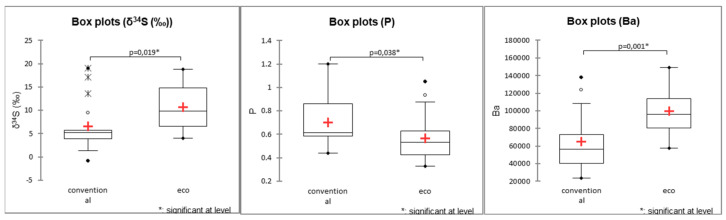
Box plots of *δ*^34^S, P, and Ba concentrations in cinnamon samples by agricultural production method. The red “+” symbol indicates the mean value for each group.

To confirm significant differences between organic and conventional samples, we applied the Mann–Whitney test to 17 conventional and 19 organic cinnamon samples, further validating the separation based on elemental and isotopic profiles. The Mann–Whitney test identified significant differences (*p* < 0.05) in *δ*^34^S, P, and Ba between organic and conventional cinnamon (Figure 13).

Organic cinnamon production shows statistically significantly higher average concentrations of Ba (99.6 mg/kg) and *δ*^34^S values (10.7‰), while conventional cinnamon production shows higher average concentrations of P (701 mg/kg), likely due to P-rich fertilizers. Similar trends were observed in paprika, where *δ*^34^S helped differentiate organic and conventional production, with organic samples showing higher *δ*^34^S values than conventional ones. This contrasts with the findings of Sinkovič et al. [67] in chicory, where organically produced samples exhibited lower *δ*^34^S values compared to conventionally fertilized ones. Schmidt et al. [52] reported *δ*^34^S as more indicative of origin than fertilizer type. Barium content also differed geographically (Sri Lanka—Vietnam; Indonesia—Vietnam), while P remained consistent, reinforcing its potential as a marker of production method. While *δ*^34^S can provide valuable insights for distinguishing farming practices, its strong sensitivity to geographic origin and environmental conditions limits its reliability as a standalone marker. Variations in *δ*^34^S arise from differences in sulfur sources (such as elemental sulfur commonly used in organic farming), soil sulfur cycling, and other environmental factors, which can cause *δ*^34^S values to fluctuate independently of farming method. Therefore, *δ*^34^S is most effective when combined with complementary markers like P to enhance the accuracy of agricultural authentication.

Misclassifications, such as C16, underline the challenges of relying solely on elemental and isotopic markers for differentiation. They highlight the need for additional analytical methods, such as FTIR, to improve classification accuracy and address borderline cases. No significant differences in *δ*^15^N values were found between organic and conventional cinnamon, indicating that *δ*^15^N alone is not a reliable marker for agricultural production methods across all food products. However, geographical variations in *δ*^15^N were observed, particularly between Indonesian and Sri Lankan cinnamon, reinforcing the need to account for origin in interpreting isotopic data.

## 4. Conclusions

This study highlights the effectiveness of an integrated analytical strategy—combining isotopic, elemental, spectroscopic, and antioxidant profiling with advanced chemometric modeling—for the authentication and compositional assessment of paprika and cinnamon. Significant differences were found between the two spices, with cinnamon, especially Sri Lankan cassia, showing much higher antioxidant activity (AA) and total phenolic content (TPC) than paprika. The strong positive correlation between AA and TPC in cinnamon highlights the impact of species on bioactive compounds. While cinnamon’s chemical composition varied notably by geographical origin, paprika exhibited relatively little variation across origins.

Stable isotope and elemental profiling enabled the robust classification of geographical origin and agricultural production methods for paprika and cinnamon. For paprika, origin prediction achieved 90% accuracy using key markers such as *δ*^34^S, Mg, Sr, Cs, Rb, V, *δ*^13^C, Fe, Al, P, S, and Ba, effectively differentiating samples from Hungary, Serbia, and Spain. Organic paprika samples were characterized by elevated *δ*^15^N, *δ*^34^S, and Zn, while conventional samples showed significantly higher concentrations of Na, Al, Cr, Pb, and V, consistent with synthetic fertilizers and agrochemicals.

In cinnamon, classification by origin reached 89% accuracy, separating samples from Sri Lanka, Madagascar, and Indonesia using Rb, Cu, Ca, Se, *δ*^13^C, Na, *δ*^34^S, Cs, Mn, *δ*^15^N, Al, V, Cd, and Fe as key markers. The differentiation of agricultural production methods yielded 95% accuracy, identifying *δ*^34^S, Ba, and P as the most influential markers. While organic cinnamon typically exhibited higher Ba and *δ*^34^S values, the interpretation of *δ*^34^S was complicated by its strong dependence on geographical origin. Furthermore, *δ*^15^N did not reliably distinguish between organic and conventional cinnamon, highlighting the need to interpret isotopic data in the context of both origin and farming practices.

Lower classification and overlapping of the statistical evaluation according to origin were further supported by FTIR analysis, indicating possible adulteration. FTIR added complementary insights, effectively distinguishing Ceylon from cassia cinnamon and revealing potential adulteration in paprika. Further, in cinnamon, distinct bands at 780 and 750 cm^−1^ enabled species identification and reflected differences in farming practices, corroborating the AA and TPC results.

The distinct profiles observed across species, geographical origins, and production methods demonstrate the necessity of integrated multi-marker approaches for reliable spice authentication. However, ensuring adequate numerical representation of each product type, along with confirming their provenance and composition, is essential to strengthen the model’s reliability and applicability across real-world scenarios. This was not fully achievable in the present study and will therefore be prioritized in future work by expanding the sample size and applying rigorous selection criteria to better capture the variability of the products under investigation.

## Figures and Tables

**Table 1:** Range of the concentrations of elements in paprika samples.

Elements	Paprika (mg/kg)
Macroelements	
Na	98.2–1540
Mg	1670–3310
P	2580–4720
S	1840–2780
K	17,400–34,300
Ca	854–3580
Microelements	
V	0.03–1.18
Cr	0.16–5.74
Mn	8.88–40.2
Fe	66.2–607
Co	0.05–1.18
Ni	0.13–3.84
Cu	7.71–13.8
Zn	13.6–32.5
Al	17.7–629
Se	0.006–0.17
Rb	5.59–41.1
Sr	3.87–33.4
Mo	0.07–1.97
Ag	0.001–0.09
Cs	0.01–0.20
Ba	0.62–7.88
Toxic elements	
Hg	0.0004–0.0032
Pb	0.01–0.54
As	0.02–0.18
Cd	0.028–0.318

**Table 2:** Range of the concentrations of elements in cinnamon samples.

Elements	Cinnamon (mg/kg)
Macroelements	
Na	6–1760
Mg	517–1440
P	327–1200
S	638–3400
K	4420–15,500
Ca	6520–35,600
Microelements	
V	0.03–3.31
Cr	0.05–4.68
Mn	21.3–598
Fe	15.4–1680
Co	0.02–0.81
Ni	0.08–6.45
Cu	2.12–12.3
Zn	5.42–27.1
Al	23.2–2390
Se	0.003–0.14
Rb	7.62–39.2
Sr	32.3–256
Mo	0.01–0.27
Ag	0.001–0.02
Cs	0.03–0.89
Ba	22.5–152
Toxic elements	
Hg	0.002–0.062
Pb	0.021–4.01
As	0.01–0.25
Cd	0.05–0.70

## Data Availability

The original contributions presented in this study are included in the article. Further inquiries can be directed to the corresponding author.

## Supplementary Materials

The following supporting information can be downloaded at https://www.mdpi.com/article/10.3390/foods14132323/s1, Table S1: Inventory of the paprika powder samples with sample designation, description, geographical origin, and agricultural method of production; Table S2: Inventory of the cinnamon samples with sample designation, description, geographical origin, and agricultural method of production; Table S3: Antioxidant Activity (AA; mg TE/g LV) and Total Phenolic Compounds (TPC; mg GA/g LV) in paprika samples extracted with 70% ethanol. Values are presented as the mean ± standard deviation; Table S4: Antioxidant Activity (AA; mg TE/g LV) and Total Phenolic Compounds (TPC; mg GA/g LV) in cinnamon samples extracted with 70% ethanol. Values are presented as the mean ± standard deviation; Table S5: Wavenumbers (cm^−1^) of identified peaks in IR spectra of paprika samples; Table S6: Correlation coefficients between IR spectra of paprika samples; Table S7: Wavenumbers (cm^−1^) of identified peaks in IR spectra of cinnamon samples; Table S8: Correlation coefficients between IR spectra of cinnamon samples; Figure S1: Box plots of elemental concentrations (Fe, Zn, As, Rb, Ba, Pb, Cs) in paprika samples by agricultural method of production where statistical differences were observed.

## Abbreviations

The following abbreviations are used in this manuscript:

AA	Antioxidant Activity
DPPH	2,2-Diphenyl-1-picrylhydrazyl
TPC	Total Phenolic Content
FTIR	Fourier-Transform Infrared Spectroscopy
IRMS	Isotope Ratio Mass Spectrometry
ICP-MS	Inductively Coupled Plasma Mass Spectrometry
PCA	Principal Component Analysis
OPLS-DA	Orthogonal Partial Least Squares Discriminant Analysis
DA	Discriminant Analysis
ED-XRF	Energy-Dispersive X-ray Fluorescence
EU	European Union
SIMCA	Soft Independent Modeling by Class Analogy

## References

[1] European Commission (2021). Results of the First Coordinated Control Plan on the Authenticity of Herbs and Spices. https://ec.europa.eu/newsroom/sante/items/727969/en.

[2] Danciu V., Hosu A., Cimpoiu C. (2018). Thin-Layer Chromatography in Spices Analysis. J. Liq. Chromatogr. Relat. Technol..

[3] Pustjens A.M., Weesepoel Y., Ruth S.M., Leadley C.E. (2016). Food Fraud and Authenticity: Emerging Issues and Future Trends. Innovation and Future Trends in Food Manufacturing and Supply Chain Technologies.

[4] Embuscado M.E. (2019). Bioactives from culinary spices and herbs: A review. J. Food Bioact..

[5] Kim E.A., Lee S.Y., Baek D.Y., Park S.-Y. (2019). A Comparison of the Nutrient Composition and Statistical Profile in Red Pepper Fruits (*Capsicum annuum* L.) Based on Genetic and Environmental Factors. Appl. Biol. Chem..

[6] Vinković T., Gluščić V., Mendaš G., Vinković Vrček I., Parađiković N., Tkalec M., Štolfa Čamagajevac I. (2018). Phytochemical Composition of Ground Paprika from the Eastern Danube Region. Poljoprivreda.

[7] Huang H., Chen R., Ma H., Yuan Z. (2018). Quality Attributes and Chemical Composition of Commercial Cinnamon Oils. Qual. Assur. Saf. Crops Foods.

[8] Velázquez R., Rodríguez A., Hernández A., Casquete R., Benito M.J., Martín A. (2023). Spice and herb frauds: Types, incidence, and detection: The state of the art. Foods.

[9] Vera D.N., Ruisánchez I., Callao M.P. (2018). Establishing Time Stability for Multivariate Qualitative Methods. Case Study: Sudan I and IV Adulteration in Food Spices. Food Control.

[10] Galvin-King P., Haughey S.A., Elliott C.T. (2020). The detection of substitution adulteration of paprika with spent paprika by the application of molecular spectroscopy tools. Foods.

[11] Blahová J., Svobodová Z. (2012). Assessment of Coumarin Levels in Ground Cinnamon Available in the Czech Retail Market. Sci. World J..

[12] National Center for Complementary and Integrative Health (NCCIH) (2024). Cinnamon: Usefulness and Safety. https://www.nccih.nih.gov/health/cinnamon.

[13] Feltes G., Ballen S.C., Steffens J., Paroul N., Steffens C. (2023). Differentiating True and False Cinnamon: Exploring Multiple Approaches for Discrimination. Micromachines.

[14] Castro R.C., Ribeiro D.S.M., Santos J.L.M., Pascoa R.N.M.J. (2023). Authentication/Discrimination, Identification, and Quantification of Cinnamon Adulterants Using NIR Spectroscopy and Different Chemometric Tools: A Tutorial to Deal with Counterfeit Samples. Food Control.

[15] U.S. Food and Drug Administration (FDA) (2024). Ground Cinnamon Products Added to FDA Public Health Alert Due to Presence of Elevated Levels of Lead. https://www.fda.gov/food/alerts-advisories-safety-information/more-ground-cinnamon-products-added-fda-public-health-alert-due-presence-elevated-levels-lead.

[16] U.S. Food and Drug Administration (FDA) (2024). FDA Public Health Alert for Additional Ground Cinnamon Product Due to Presence of Elevated Levels of Lead. https://www.fda.gov/food/alerts-advisories-safety-information/fda-alert-concerning-certain-cinnamon-products-due-presence-elevated-levels-lead.

[17] NPR (2024). Lead in Cinnamon: Where Do Things Stand, 1 Year After a Scary Recall?. https://www.npr.org/2024/10/24/nx-s1-5119336/cinnamon-lead-fda-recall-what-we-know.

[18] Everstine K., Spink J., Kennedy S. (2013). Economically Motivated Adulteration (EMA) of Food: Common Characteristics of EMA Incidents. J. Food Prot..

[19] Castell A., Arroyo-Manzanares N., López-García I., Zapata F., Viñas P. (2024). Authentication strategy for paprika analysis according to geographical origin and study of adulteration using near infrared spectroscopy and chemometric approaches. Food Control.

[20] Chao K., Dhakal S., Schmidt W.F., Qin J., Kim M., Peng Y., Huang Q. (2020). Raman and IR spectroscopic modality for authentication of turmeric powder. Food Chem..

[21] Núñez N., Vidal-Casanella O., Sentellas S., Saurina J., Núñez O. (2020). Characterization, classification and authentication of turmeric and curry samples by targeted LC-HRMS polyphenolic and curcuminoid profiling and chemometrics. Molecules.

[22] Cruz-Tirado J.P., de Franca R.L., Tumbajulca M., Barraza-Jauregui G., Barbin D.F., Siche R. (2023). Detection of cumin powder adulteration with allergenic nutshells using FT-IR and portable NIRS coupled with chemometrics. J. Food Compos. Anal..

[23] Rocamora-Rivera B., Arroyo-Manzanares N., Viñas P. (2024). Detection of adulterated oregano samples using untargeted headspace–gas chromatography–ion mobility spectrometry analysis. Foods.

[24] Schendel R.R., Pandeya P.R., Ismail B.P., Nielsen S.S. (2024). Determination of Total Phenolics and Antioxidant Capacity in Food and Ingredients. Nielsen’s Food Analysis.

[25] Gulcin İ., Alwasel S.H. (2023). DPPH Radical Scavenging Assay. Processes.

[26] Zhao T., Nakano A. (2018). Agricultural Product Authenticity and Geographical Origin Traceability. Jpn. Agric. Res. Q..

[27] Danezis G.P., Tsagkaris A.S., Camin F., Brusic V., Georgiou C.A. (2016). Food Authentication: Techniques, Trends & Emerging Approaches. TrAC Trends Anal. Chem..

[28] Krauß S., Vetter W. (2019). Stable Carbon and Nitrogen Isotope Ratios of Red Bell Pepper Samples from Germany, The Netherlands, and Spain. J. Agric. Food Chem..

[29] De Rijke E., Schoorl J.C., Cerli C., Vonhof H.B., Verdegaal S.J.A., Vivó-Truyols G., Lopatka M., Dekter R., Bakker D., Sjerps M.J. (2016). The use of δ2H and δ18O isotopic analyses combined with chemometrics as a traceability tool for the geographical origin of bell peppers. Food Chem..

[30] Brunner M., Katona R., Stefanka Z. (2010). Determination of the geographical origin of processed spice using multielement and isotopic patterns on the example of Szegedi paprika. Eur. Food Res. Technol..

[31] Rohman A., Ghazali M.A.B., Windarsih A., Irnawati, Riyanto S., Yusof F.M. (2020). Comprehensive Review on Application of FTIR Spectroscopy Coupled with Chemometrics for Authentication Analysis of Fats and Oils in Food Products. Molecules.

[32] Lixourgioti P., Goggin K.A., Zhao X., Murphy D.J., van Ruth S., Koidis A. (2022). Authentication of Cinnamon Spice Samples Using FT-IR Spectroscopy and Chemometric Classification. LWT.

[33] Brand-Williams W., Cuvelier M.E., Berset C. (1995). Use of a Free Radical Method to Evaluate Antioxidant Activity. Lebensm. Wiss. Technol..

[34] Gutfinger T. (1981). Polyphenols in Olive Oils. J. Am. Oil Chem. Soc..

[35] Lohumi S., Lee S., Cho B.K. (2015). Optimal Variable Selection for Fourier Transform Infrared Spectroscopic Analysis of Starch-Adulterated Garlic Powder. Sens. Actuators B Chem..

[36] Potočnik D., Jagodic Hudobivnik M., Mazej D., Ogrinc N. (2021). Optimization of the sample preparation method for determination of multi-elemental composition in fruit samples by ICP-MS analysis. Meas. Sens..

[37] Mudrić S., Gašić U.M., Dramićanin A.M., Ćirić I.Ž., Milojković-Opsenica D.M., Popović-Đorđević J.B., Momirović N.M., Tešić Ž.L. (2017). The polyphenolics and carbohydrates as indicators of botanical and geographical origin of Serbian autochthonous clones of red spice paprika. Food Chem..

[38] Tvrzník P., Jeřábek T., Kráčmar S., Fišera M. (2019). Changes in Phenolic Content in Ground Red Pepper (*Capsicum annuum* L.) during Storage. J. Microbiol. Biotechnol. Food Sci..

[39] Medina-Juárez L.Á., Molina-Quijada D.M.A., Del Toro Sánchez C.L., González-Aguilar G.A., Gámez-Meza N. (2012). Antioxidant Activity of Peppers (*Capsicum annuum* L.) Extracts and Characterization of Their Phenolic Constituents. Interciencia.

[40] Rudan Tasič D., Klofutar C., Škerjanc J., Šmalc A., Golob T. (2007). Fizikalnokemijske Metode v Živilstvu [Physicochemical Methods in Food Science].

[41] Dominguez-Martinez I., Meza-Marquez O., Osorio-Revilla G., Proal-Najera J., Gallardo-Velázquez T. (2014). Determination of Capsaicin, Ascorbic Acid, Total Phenolic Compounds and Antioxidant Activity of *Capsicum annuum* L. var. Serrano by Mid-Infrared Spectroscopy (Mid-FTIR) and Chemometric Analysis. J. Korean Soc. Appl. Biol. Chem..

[42] Lv J., Huang H., Yu L., Whent M., Niu Y., Shi H., Wang T.Y., Luthria D., Charles D., Yu L.L. (2012). Phenolic composition and nutraceutical properties of organic and conventional cinnamon and peppermint. Food Chem..

[43] Horn B., Esslinger S., Pfister M., Fauhl-Hassek C., Riedl J. (2018). Non-Targeted Detection of Paprika Adulteration Using Mid-Infrared Spectroscopy and One-Class Classification. Food Chem..

[44] Galvin-King P., Haughey S.A., Elliott C.T. (2017). Herb and Spice Fraud: The Drivers, Challenges, and Detection. Food Control.

[45] Palacios-Morillo A., Jurado J.M., Alcazar A., Pablos F. (2014). Geographical Characterization of Spanish PDO Paprika by Multivariate Analysis of Multielemental Content. Talanta.

[46] Garcia E., Cabrera C., Lorenz M.L., Lopez M.C. (2000). Chromium Levels in Spices and Aromatic Herbs. Sci. Total Environ..

[47] Lopez F.F., Cabrera C., Lorenzo M.L., Lopez M.C. (2000). Aluminum Levels in Spices and Aromatic Herbs. Sci. Total Environ..

[48] Ördög A., Poor P., Štajner D.I., Popović B.M., Batori Z., Tari I. (2018). Comparison of the Mineral Content of Processed Spice Samples of Sweet and Hot Paprika from the Szeged Region. J. Elementol..

[49] Mahne Opatić A.M., Nečemer M., Lojen S., Vidrih R. (2017). Stable Isotope Ratio and Elemental Composition Parameters in Combination with Discriminant Analysis Classification Model to Assign Country of Origin to Commercial Vegetables—A Preliminary Study. Food Control.

[50] Fiamegos Y., Dumitrascu C., Papoci S., de la Calle M.B. (2021). Authentication of PDO Paprika Powder (Pimentón de la Vera) by Multivariate Analysis of the Elemental Fingerprint Determined by ED-XRF—A Feasibility Study. Food Control.

[51] Fiamegos Y., Papoci S., Dumitrascu C., Ghidotti M., Zdiniakova T., Ulberth F., de la Calle Guntiñas M.B. (2021). Are the Elemental Fingerprints of Organic and Conventional Food Different? ED-XRF as Screening Technique. J. Food Compos. Anal..

[52] Schmidt H.L., Rossmann A., Voerkelius S., Schnitzler W.H., Georgi M., Grassmann J., Zimmermann G., Winkler R. (2005). Isotope Characteristics of Vegetables and Wheat from Conventional and Organic Production. Isot. Environ. Health Stud..

[53] Flores P., Fenoll J., Hellín P. (2007). The Feasibility of Using δ^15^N and δ^13^c Values for Discriminating between Conventionally and Organically Fertilized Pepper (*Capsicum annuum* L.). J. Agric. Food Chem..

[54] Perez-Lopez A.J., Lopez-Nicolas J.M., Nuñez-Delicado E., Amor F.M., Carbonell-Barrachina A.A. (2007). Effects of Agricultural Practices on Color, Carotenoid Composition, and Mineral Contents of Sweet Peppers, cv. Almuden. J. Agric. Food Chem..

[55] Santos Lopes J., Sales de Lima A.B., Ribeiro da Cruz Cangussu R., Viana da Silva M., Passini Barbosa Ferrão S., Soares Santos L. (2022). Application of Spectroscopic Techniques and Chemometric Methods to Differentiate Between True Cinnamon and False Cinnamon. Food Chem..

[56] Li Y.Q., Kong D.X., Wu H. (2013). Analysis and Evaluation of Essential Oil Components of Cinnamon Barks Using GC–MS and FTIR Spectroscopy. Ind. Crops Prod..

[57] Yasmin J., Ahmed M.R., Lohumi S., Wakholi C., Lee H., Mo C., Cho B.K. (2019). Rapid Authentication Measurement of Cinnamon Powder Using FT-NIR and FT-IR Spectroscopic Techniques. Qual. Assur. Saf. Crops Foods.

[58] Silva Bruni A.R., Oliveira V.M.A.T., Fernandez A.S.T., Sakai O.A., Marco P.H., Valderrama P. (2021). Attenuated Total Reflectance Fourier Transform (ATR-FTIR) Spectroscopy and Chemometrics for Organic Cinnamon Evaluation. Food Chem..

[59] Shumaila G., Safdar M. (2009). Proximate Composition and Mineral Analysis of Cinnamon. Pak. J. Nutr..

[60] Özcan M.M., Akbulut M. (2007). Estimation of Minerals, Nitrate, and Nitrite Contents of Medicinal and Aromatic Plants Used as Spices, Condiments, and Herbal Tea. Food Chem..

[61] Goncalves L.L., Fernandes T., Bernardo M.A., Brito J.A. (2018). Assessment of Human Health Risk of Toxic Elements Due to Cinnamon Ingestion in the Diet. Biol. Trace Elem. Res..

[62] Krejpcio Z., Krol E., Sionkowski S. (2006). Evaluation of Heavy Metal Contents in Spices and Herbs Available on the Polish Market. Pol. J. Environ. Stud..

[63] Singh V., Garg A.N. (2006). Availability of Essential Trace Elements in Indian Cereals, Vegetables, and Spices Using INAA and the Contribution of Spices to Daily Dietary Intake. Food Chem..

[64] Tokalioglu S. (2012). Determination of Trace Elements in Commonly Consumed Medicinal Herbs by ICP-MS and Multivariate Analysis. Food Chem..

[65] Primožič S. (2024). Determining the Authenticity of Cinnamon (*Cinnamomum* spp.) and Ground Pepper (*Capsicum annuum*) on the Slovenian Market. Master’s Thesis.

[66] Sewenig S., Hener U., Mosandl A. (2003). Online Determination of ^2^H/^1^H and ^13^C/^12^C Isotope Ratios of Cinnamaldehyde from Different Sources Using Gas Chromatography Isotope Ratio Mass Spectrometry. Eur. Food Res. Technol..

[67] Sinkovič L., Nečemer M., Ogrinc N., Žnidarčič D., Stopar D., Vidrih R., Meglič V. (2020). Parameters for Discrimination between Organic and Conventional Production: A Case Study for Chicory Plants (*Cichorium intybus* L.). Food Chem. Toxicol..

