# Supplementary material for: Assessing the Authenticity and Quality of Paprika (Capsicum annuum) and Cinnamon (Cinnamomum spp.) in the Slovenian Market: A Multi-Analytical and Chemometric Approach
Source: Foods. 2025 Jun 30;14(13):2323. doi: 10.3390/foods14132323 (PMC12248926; doi:10.3390/foods14132323)
Supplement: Supplementary file 1 [file foods-14-02323-s001.zip › foods-3688291-supplementary.pdf]

Supplementary Material

Assessing the Authenticity and Quality of Paprika (*Capsicum annuum*) and Cinnamon (*Cinnamomum spp.*) in the Slovenian Market: A Multi-Analytical Approach

Sabina Primožič[1.+]. Cathrine Terro[2.3.+]. Lidija Strojnik[2]. Nataša Poklar Ulrih[1] and Nives Ogrinc[2.3*]

[1] *Department of Food Science and Technology. Biotechnical Faculty. University of Ljubljana. Jamnikarjeva 101. 1000 Ljubljana. Slovenia; sabina.primozic@hotmail.com. Natasa.Poklar@bf.uni-lj.si*

[2] *Department of Environmental Sciences. Jožef Stefan Institute. Jamova 39. 1000 Ljubljana. Slovenia; cathrine.terro@ijs.si. Lidija.strojnik@ijs.si. nives.ogrinc@ijs.si*

[3] *International Postgraduate School Jožef Stefan Institute. Jamova 39. 1000 Ljubljana. Slovenia; cathrine.terro@ijs.si. nives.ogrinc@ijs.si*

[+] These authors contributed equally to this work.

*Correspondence: nives.ogrinc@ijs.si; Tel.: 00386 1 5885387

Table S1. Inventory of the paprika powder samples with sample designation. description. geographical origin and agricultural method of production

Sample ID	Description	Origin	Method of production
P1	Sweet paprika	Hungary	Conventional
P2	Delicatessen paprika	Hungary	Conventional
P3	Sweet ground paprika	Serbia	Conventional
P4	Hot ground paprika	Serbia	Conventional
P5	Tucana – crushed red hot paprika	Serbia	Conventional
P6	Hot ground paprika	Hungary	Conventional
P7	Smoked hot ground paprika	Spain	Conventional
P8	Sweet ground paprika	Spain	Conventional
P9	Smoked sweet ground paprika	Spain	Conventional
P10	Sweet ground paprika	Spain	Conventional
P11	Smoked hot ground paprika	Spain	Conventional
P12	Pounded paprika	Hungary	Conventional

P13	Smoked ground paprika	Spain	Conventional
P14	Hot ground paprika	Hungary	Conventional
P15	Sweet ground paprika	Peru	Conventional
P16	Hot ground paprika	India	Conventional
P17	Hot paprika	Spain. Hungary. China. India (/)	Conventional
P18	Smoked hot paprika	Spain	Conventional
P19	Hot ground paprika	/	Conventional
P20	Smoked sweet paprika	Spain	Conventional
P21	Bio pekoča paprika	Spain	Organic
P22	Bio smoked paprika	Spain	Organic
P23	Bio sweet paprika	Spain	Organic
P24	Paprika powder	Spain	Conventional
P25	Eco sweet paprika	Spain	Organic
P26	Pounded sweet paprika	Spain	Conventional
P27	Paprika pieces/ fragments	Spain	Conventional
P28	Pounded hot paprika	Spain	Conventional
P29	Sweet ground paprika	China	Conventional
P30	Smoked sweet paprika	Spain	Conventional
P31	Hot ground paprika	Spain	Conventional
P32	Sweet paprika	/	Conventional
P33	Smoked paprika	/	Conventional
P34	Hot paprika	/	Conventional
P35	Sweet ground paprika	Peru	Conventional
P36	Smoked ground paprika	China	Conventional
P37	Sweet ground paprika	Croatia	Conventional
P38	Sweet red paprika	Spain	Conventional
P39	Leskovačka sweet ground paprika	Serbia	Conventional

P40	Paprika	Hungary 50 %. Serbia 50 % (/)	Conventional
P41	Sweet ground paprika	/	Conventional
P42	Sweet ground paprika	Spain	Conventional
P43	Bio hot ground paprika	Agriculture in the EU (/)	Organic
P44	Smoked ground paprika	Spain	Conventional
P45	Sweet paprika	Spain	Conventional

Table S2. Inventory of the cinnamon samples with sample designation. description. geographical origin and agricultural method of production

Sample ID	Description	Origin	Method of production
C1	"Bio" ground cinnamon	Vietnam	Organic
C2	"Bio" Ceylon cinnamon sticks	Agriculture outside the EU (/)	Organic
C3	"Bio" Ceylon cinnamon sticks	Agriculture outside the EU (/)	Organic
C4	"Eco" Ceylon ground cinnamon	Sri Lanka	Organic
C5	"Bio" Ceylon ground cinnamon	Sri Lanka	Organic
C6	"Bio" ground cinnamon cassia. *C. cassia*	Indonesia	Organic
C7	"Bio" Ceylon ground cinnamon	EU/non-EU agriculture (/)	Organic
C8	Ground cinnamon	Indonesia	Conventional
C9	Ceylon ground cinnamon	Sri Lanka	Conventional
C10	Ceylon ground cinnamon	Sri Lanka	Conventional
C11	Ceylon ground cinnamon	Sri Lanka	Conventional
C12	Ground cinnamon cassia	Indonesia	Conventional
C13	Ceylon cinnamon powder (Cinnamomi ceylon cortex pulvis)	Madagascar	Conventional
C14	Ceylon cinnamon	/	Conventional
C15	"Bio" Ceylon cinnamon	Madagascar	Organic
C16	Ceylon cinnamon	Madagascar	Conventional
C17	Ground cinnamon	/	Conventional

C18	Ground cinnamon	China	Conventional
C19	Ground cinnamon	Indonesia	Conventional
C20	"Bio" Ceylon cinnamon	Madagascar	Organic
C21	Ground cinnamon cassia	Indonesia	Conventional
C22	"Bio" Ceylon cinnamon in the threshold	Agriculture outside the EU (/)	Organic
C23	"Eco" ground cinnamon from Ceylon	Sri Lanka	Organic
C24	"Bio" ground cinnamon	Agriculture outside the EU (/)	Organic
C25	Ground cinnamon	India	Conventional
C26	Ground cinnamon	Indonesia. Sri Lanka. China. Madagascar (/)	Conventional
C27	Ground cinnamon	/	Conventional
C28	Ground cinnamon	Indonesia	Conventional
C29	Ground cinnamon	/	Conventional
C30	"Bio" ground cinnamon Ceylon. *C. verum*	Sri Lanka	Organic
C31	Ground cinnamon	/	Conventional
C32	"Bio" Ceylon cinnamon sticks	Agriculture outside the EU (/)	Organic
C33	"Bio" ground cinnamon (*C. cassia*)	Vietnam	Organic
C34	"Bio" Ceylon ground cinnamon	Madagascar	Organic
C35	"Bio" cinnamon in the threshold	Agriculture outside the EU (/)	Organic
C36	Ground cinnamon	Indonesia	Conventional
C37	Cinnamon Ceylon bark – sticks	Sri Lanka	Conventional
C38	"Bio" cinnamon sticks. Ceylon (true) cinnamon	Sri Lanka	Organic
C39	Whole cinnamon – sticks	/	Conventional
C40	"Bio" whole Ceylon cinnamon sticks	Agriculture outside the EU (/)	Organic
C41	Cinnamon in the crust (*C. cassia*) sticks	Indonesia	Conventional
C42	Cinnamon and C. zeylanicum Blume sticks	Sri Lanka	Conventional
C43	Cinnamon sticks	Sri Lanka	Conventional
C44	Whole Ceylon cinnamon sticks	Sri Lanka	Conventional
C45	Cinnamon	Sri Lanka	Conventional
C46	Cinnamon	/	Conventional

Table S3. Antioxidant Activity (AA; mg TE/g LV) and Total Phenolic Compounds (TPC; mg GA/g LV) in paprika samples extracted with 70% ethanol. Values are presented as the mean ± standard deviation.

Sample ID	Antioxidant Activity (AA)	Total Phenolic Compounds (TPC)
P1	4.65 ± 0.07	14.7 ± 0.1
P2	4.52 ± 0.05	12.7 ± 0.2
P3	3.32 ± 0.10	11.0 ± 0.3
P4	3.95 ± 0.06	12.5 ± 0.1
P10	4.13 ± 0.10	13.0 ± 0.3
P12	2.87 ± 0.06	9.4 ± 0.2
P15	4.60 ± 0.07	12.9 ± 0.2
P16	2.36 ± 0.08	8.1 ± 0.1
P17	3.55 ± 0.08	10.6 ± 0.2
P18	3.52 ± 0.05	12.9 ± 0.1
P20	4.30 ± 0.05	11.2 ± 0.1
P21	3.40 ± 0.09	12.2 ± 0.3
P22	3.93 ± 0.04	15.0 ± 0.1
P23	4.25 ± 0.07	13.0 ± 0.1
P25	4.14 ± 0.06	15.4 ± 0.1
P26	3.45 ± 0.06	13.7 ± 0.1
P27	4.31 ± 0.06	16.2 ± 0.2
P28	3.27 ± 0.06	13.8 ± 0.1
P29	3.45 ± 0.08	10.7 ± 0.2
P31	3.52 ± 0.09	11.8 ± 0.1
P36	3.97 ± 0.09	12.2 ± 0.2
P37	3.90 ± 0.09	13.7 ± 0.2
P39	3.06 ± 0.08	8.3 ± 0.2
P42	2.43 ± 0.07	7.5 ± 0.2

Table S4. Antioxidant Activity (AA; mg TE/g LV) and Total Phenolic Compounds (TPC; mg GA/g LV) in cinnamon samples extracted with 70% ethanol. Values are presented as the mean ± standard deviation.

Sample ID	Antioxidant activity (AA)	Total Phenolic Compounds (TPC)
C5	55 ± 1	33 ± 1
C6	138 ± 1	74 ± 1
C9	97 ± 1	86 ± 1
C10	65 ± 1	70 ± 1
C11	56 ± 1	40 ± 0.2
C12	158 ± 1	177 ± 1
C13	172 ± 1	103 ± 1
C15	106 ± 1	79 ± 1
C16	146 ± 1	83 ± 1
C20	125 ± 1	81 ± 0.3
C21	341 ± 1	247 ± 1
C23	53 ± 1	40 ± 1
C24	404 ± 1	254 ± 1
C27	143 ± 1	79 ± 1
C28	157 ± 1	108 ± 1
C33	152 ± 1	92 ± 0.3
C37	165 ± 1	136 ± 1
C38	111 ± 1	67 ± 1
C42	142 ± 1	82 ± 0.1

Table S5. Wavenumbers (cm^{-1}) of identified peaks in IR spectra of paprika samples

**Sample ID**	**FUNCTIONAL GROUP AREA**							**FINGERPRINT AREA**				
	O-H	C-H	C-H	C≡C	C=O	C=O	C=C<br>C=O	C-H	C-O	C-O<br>C-C	C-O<br>C-H	C-H<br>O-H
	Peaks (cm^{-1})											
P1	3280	2924	2854	2111	1739	1712	1623	1377	1237	1147	1028	816
P2	3280	2924	2854	2116	1741	/	1627	1376	1237	1149	1027	817
P3	3280	2924	2854	2107	1742	/	1623	1377	1237	1151	1028	817
P4	3280	2924	2854	2104	1741	/	1623	1377	1236	1153	1027	816
P5	3280	2924	2854	2100	1742	/	1623	1377	1237	1147	1027	816
P6	3281	2924	2854	2104	1742	/	1622	1377	1236	1155	1028	815
P7	3281	2924	2854	2107	1741	1711	1616	1378	1237	1151	1027	816
P8	3279	2924	2854	2103	1741	/	1622	1377	1236	1146	1028	816
P9	3280	2924	2854	2105	1742	/	1623	1377	1236	1148	1028	815
P10	3280	2924	2854	2108	/	1710	1616	/	1240	/<br>1146	1027	/<br>815
P11	3280	2924	2854	2101	/	1710	1624	1378	1240	/<br>1156	1028	816
P12	3279	2924	2854	2100	1743	/	1625	1378	1236	1147	1028	/<br>814
P13	3280	2924	2854	2112	1741	1710	1623	1378	1239	1153	1027	/<br>815
P14	3280	2924	2854	2109	/	1711	1623	1378	1237	1146	1028	816
P15	3281	2927	2854	2104	1742	/	1622	1377	1236	1152	1027	816
P16	3280	2924	2854	2100	1742	/	1625	1378	1236	1153	1028	/<br>815
P17	3282	2924	2854	2100	1742	/	1622	1377	1236	1155	1028	815

P18	3282	2924	2854	2108	/	1710	1623	1378	1239	1146	1028	816
P19	3281	2924	2854	2100	1742	1710	1626	1378	1238	1156	1028	/<br>812
P20	3281	2924	2854	2116	1740	1711	1623	1378	1238	1147	1027	816
P21	3281	2924	2854	2113	1742	/	1615	1377	1236	1145	1027	816
P22	3280	2924	2854	2115	/	1710	1624	1377	1241	1147	1028	816
P23	3281	2924	2854	2104	1736	1711	1623	1377	1237	/<br>1144	1028	816
P24	3280	2924	2854	2109	/	1710	1622	1378	1240	1147	1027	815
P25	3280	2924	2854	2112	1741	/	1622	1377	1236	1147	1027	817
P26	3279	2925	2855	2104	/	1709	1623	1384	1238	1146	1031	816
P27	3276	2925	2855	2103	1734	/	1619	1375	1238	1147	1027	817
28	3281	2924	2854	2115	1736	1710	1623	1379	1239	1148	1028	816
P29	3278	2924	2854	2115	1741	/	1620	1378	1238	1147	1027	813
P30	3280	2924	2854	2112	/	1710	1624	1378	1240	/<br>1143	1027	815
P31	3281	2924	2854	2108	1740	/	1623	1381	1238	1155	1028	815
P32	3281	2923	2854	2103	1741	/	1622	1378	1238	1155	1027	815
P33	3281	2924	2854	2100	1743	/	1622	1377	1237	1155	1027	815
P34	3280	2924	2854	2102	1743	/	1623	1378	1237	1153	1028	815
P35	3281	2924	2854	2116	1741	/	1622	1377	1236	1151	1027	816
P36	3281	2924	2854	2115	1742	/	1621	1377	1237	1153	1027	815
P37	3281	2924	2854	2108	/	1710	1623	1377	1239	1149	1028	816
P38	3281	2924	2854	2104	1742	/	1622	1377	1237	1153	1027	815
P39	3283	2924	2854	2108	1741	1710	1635	1378	1238	1147	1028	815
P40	3280	2924	2854	2100	1736	1712	1623	1377	1236	1147	1027	816
P41	3281	2923	2854	2105	1743	/	1622	1377	1237	1156	1026	815

P42	3280	2924	2854	2102	1744	/	1625	1377	1237	1155	1027	814
P43	3277	2924	2854	2115	1737	/	1625	1376	1238	1148	1027	816
P44	3281	2924	2854	2104	1742	/	1622	1377	1236	1153	1027	815
P45	3281	2924	2854	2113	1742	1711	1623	1378	1238	1155	1027	816

Table S6. Correlation coefficients between IR spectra of paprika samples

ID	P23	P25	P8	P38	P42	P45	P1	P3	P39	P15	P35	P29	P4	P5	P6	P16	P28	P31	P21	P9	P20	P7	P10	P11	P14	P18	P26	P30	P37
**P23**	1	0.969	0.969	0.923	0.840	0.914	0.979	0.939	0.952	0.916	0.940	0.954	0.930	0.949	0.936	0.926	0.948	0.960	0.977	0.944	0.958	0.959	0.964	0.949	0.989	0.978	0.944	0.913	0.957
**P25**		1	0.986	0.960	0.898	0.907	0.961	0.976	0.932	0.957	0.972	0.978	0.966	0.979	0.969	0.947	0.914	0.971	0.986	0.974	0.933	0.953	0.913	0.880	0.958	0.929	0.890	0.843	0.927
**P8**			1	0.970	0.902	0.924	0.979	0.980	0.954	0.966	0.979	0.988	0.976	0.988	0.976	0.964	0.929	0.979	0.986	0.985	0.950	0.961	0.932	0.905	0.96	0.943	0.904	0.869	0.949
**P38**				1	0.972	0.960	0.960	0.991	0.958	0.995	0.993	0.960	0.993	0.987	0.996	0.988	0.922	0.974	0.954	0.994	0.950	0.971	0.869	0.852	0.921	0.902	0.827	0.856	0.938
**P42**					1	0.933	0.888	0.957	0.907	0.975	0.960	0.885	0.962	0.942	0.965	0.964	0.853	0.918	0.880	0.957	0.892	0.925	0.756	0.749	0.829	0.812	0.706	0.778	0.867
**P45**						1	0.963	0.940	0.982	0.959	0.959	0.907	0.952	0.936	0.963	0.970	0.971	0.955	0.913	0.953	0.984	0.987	0.893	0.908	0.925	0.937	0.850	0.942	0.973
**P1**							1	0.962	0.984	0.955	0.968	0.964	0.964	0.969	0.968	0.964	0.974	0.978	0.975	0.971	0.988	0.985	0.962	0.956	0.986	0.981	0.933	0.940	0.988
**P3**								1	0.948	0.991	0.994	0.965	0.995	0.991	0.911	0.978	0.911	0.972	0.969	0.994	0.943	0.963	0.874	0.852	0.930	0.905	0.837	0.839	0.933
**P39**									1	0.950	0.959	0.943	0.952	0.957	0.964	0.975	0.980	0.964	0.946	0.962	0.991	0.987	0.940	0.948	0.965	0.968	0.905	0.955	0.986
**P15**										1	0.996	0.948	0.995	0.981	0.981	0.983	0.916	0.969	0.949	0.993	0.946	0.969	0.856	0.843	0.911	0.895	0.814	0.847	0.934
**P35**											1	0.962	0.995	0.986	0.995	0.983	0.929	0.979	0.964	0.996	0.956	0.977	0.884	0.870	0.935	0.918	0.845	0.865	0.947
**P29**												1	0.959	0.981	0.967	0.956	0.920	0.973	0.977	0.971	0.933	0.946	0.932	0.894	0.960	0.929	0.908	0.856	0.934
**P4**													1	0.988	0.994	0.982	0.917	0.975	0.959	0.994	0.948	0.969	0.873	0.856	0.927	0.906	0.835	0.851	0.941
**P5**														1	0.989	0.980	0.919	0.976	0.975	0.991	0.946	0.963	0.899	0.873	0.946	0.919	0.864	0.853	0.939
**P6**															1	0.988	0.933	0.981	0.964	0.995	0.958	0.978	0.886	0.869	0.934	0.917	0.848	0.868	0.947
**P16**																1	0.938	0.970	0.945	0.987	0.960	0.975	0.885	0.878	0.930	0.918	0.846	0.882	0.950
**P28**																	1	0.957	0.928	0.928	0.988	0.979	0.959	0.969	0.966	0.980	0.937	0.976	0.988
**P31**																		1	0.965	0.979	0,966	0.980	0.931	0.913	0.963	0.949	0.904	0.902	0.964
**P21**																			1	0.970	0.947	0.955	0.937	0.907	0.971	0.949	0.918	0.869	0.940
**P9**																				1	0.954	0.974	0.890	0.874	0.940	0.920	0.855	0.863	0.946
**P20**																					1	0,992	0,949	0,959	0,971	0,979	0,915	0,967	0,994
**P7**																						1	0.932	0.933	0.963	0.966	0.896	0.942	0.983
**P10**																							1	0.985	0.985	0.986	0.986	0.949	0.961
**P11**																								1	0.974	0.988	0.971	0.978	0.970
**P14**																									1	0.991	0.966	0.941	0.975
**P18**																										1	0.966	0.968	0.983
**P26**																											1	0.920	0.928
**P30**																												1	0.972
**P37**																													1

Table S7. Wavenumbers (cm^{-1}) of identified peaks in IR spectra of cinnamon samples

Sample ID	FUNCTIONAL GROUP AREA							FINGERPRINT AREA			
	O-H	C-H	*C≡C*	C=O	C=C<br>C=O	C=C	C=C	C-O	COO<br>O-H	C-O	C-H
	Peaks (cm^{-1})										
C1	3280	2923	2100	/	1604	1516	1443	1316	1244	1016	780<br>751
C2	3289	2921	2115	1731	1607	1516	1447	1318	1243	1019	782<br>755
C3	3283	2920	2108	1730	1606	1514	1436	1318	1243	1019	781<br>760
C4	3318	2920	2105	1730	1607	1515	1436	1318	1242	1019	782<br>760
C5	3281	2920	2102	1731	1606	1509	1438	1318	1243	1018	781<br>760
C6	3285	2923	2117	1728	1606	1516	1447	1317	1247	1012	780<br>750
C7	3262	2923	2111	/	1606	1520	1443	1318	/	1015	782<br>/
C8	3268	2920	2116	/	1607	1517	1442	1316	1247	1014	779<br>757
C9	3284	2921	2117	1731	1606	1517	1438	1318	1244	1015	781<br>762
C10	3282	2920	2100	1717	1606	1515	1441	1317	1244	1019	781<br>756
C11	3284	2922	2115	1712	1607	1515	1439	1317	1242	1015	781<br>760

C12	3282	2923	2108	/	1606	1516	1441	1317	1245	1015	779<br>763
C13	3267	2919	2104	1729	1606	1518	1439	1318	1245	1015	781<br>763
C14	3281	2919	2107	1731	1607	1517	1441	1318	1244	1012	781<br>761
C15	3264	2921	2107	1728	1606	1516	1441	1317	1243	1015	781<br>762
C16	3265	2920	2105	1730	1607	1516	1436	1317	1244	1016	781<br>762
C17	3281	2921	2104	1716	1606	1517	1436	1316	1243	1015	779<br>759
C18	3283	2923	2108	1728	1607	1516	1442	1317	1245	1011	780<br>754
C19	3268	2922	2101	/	1606	1518	1441	1315	1247	1013	779<br>761
C20	3282	2920	2108	1731	1606	1516	1436	1318	1243	1016	781<br>760
C21	3268	2920	2101	/	1606	1518	1441	1314	1247	1013	779<br>752
C22	3284	2920	2114	1732	1607	1509	1438	1318	1240	1020	782<br>757
C23	3283	2921	2116	1731	1607	1514	1436	1318	1241	1018	782<br>755
C24	3269	2920	2101	/	1605	1518	1439	1314	1247	1013	779<br>752
C25	3280	2921	2101	1715	1606	1516	1441	1317	1245	1016	781

											755
C26	3265	2921	2112	1727	1606	1517	1436	1316	1244	1014	/<br>759
C27	3271	2919	2104	1730	1606	1516	1436	1317	1244	1015	780<br>755
C28	3268	2920	2110	1716	1606	1517	1437	1317	1246	1015	780<br>754

Table S8. Correlation coefficients between IR spectra of cinnamon samples

**Sample ID**	**C4**	**C5**	**C23**	**C30**	**C38**	**C15**	**C20**	**C34**	**C9**	**C10**	**C11**	**C37**	**C42**	**C44**	**C13**	**C16**	**C33**	**C6**	**C12**	**C21**	**C41**
**C4**	1	0.956	0.981	0.921	0.927	0.943	0.956	0.945	0.943	0.938	0.930	0.878	0.925	0.936	0.916	0.922	0.893	0.925	0.908	0.897	0.762
**C5**		1	0.978	0.936	0.952	0.926	0.938	0.921	0.966	0.944	0.954	0.903	0.956	0.967	0.936	0.941	0.911	0.903	0.923	0.900	0.784
**C23**			1	0.936	0.944	0.948	0.960	0.944	0.963	0.953	0.946	0.897	0.947	0.955	0.935	0.942	0.911	0.928	0.926	0.907	0.780
**C30**				1	0.930	0.966	0.971	0.967	0.970	0.968	0.953	0.954	0.946	0.951	0.984	0.987	0.915	0.925	0.975	0.963	0.837
**C38**					1	0.912	0.914	0.900	0.946	0.917	0.921	0.915	0.971	0.959	0.920	0.930	0.917	0.893	0.917	0.885	0.812
**C15**						1	0.987	0.987	0.962	0.962	0.935	0.928	0.930	0.936	0.969	0.971	0.891	0.934	0.962	0.962	0.800
**C20**							1	0.988	0.963	0.967	0.943	0.917	0.925	0.935	0.970	0.971	0.897	0.936	0.954	0.951	0.791
**C34**								1	0.957	0.961	0.931	0.921	0.917	0.928	0.966	0.966	0.887	0.933	0.953	0.957	0.794
**C9**									1	0.966	0.955	0.929	0.956	0.964	0.973	0.976	0.921	0.920	0.960	0.935	0.798
**C10**										1	0.971	0.929	0.935	0.941	0.968	0.974	0.926	0.951	0.969	0.950	0.811
**C11**											1	0.901	0.934	0.938	0.945	0.956	0.916	0.931	0.944	0.913	0.772
**C37**												1	0.940	0.940	0.945	0.949	0.930	0.920	0.957	0.964	0.922
**C42**													1	0.977	0.941	0.948	0.924	0.903	0.948	0.923	0.826
**C44**														1	0.947	0.949	0.934	0.914	0.945	0.930	0.836
**C13**															1	0.987	0.904	0.910	0.976	0.968	0.821
**C16**																1	0.907	0.918	0.977	0.963	0.819
**C33**																	1	0.965	0.918	0.887	0.869
**C6**																		1	0.923	0.913	0.849
**C12**																			1	0.977	0.831
**C21**																				1	0.848
**C41**																					1

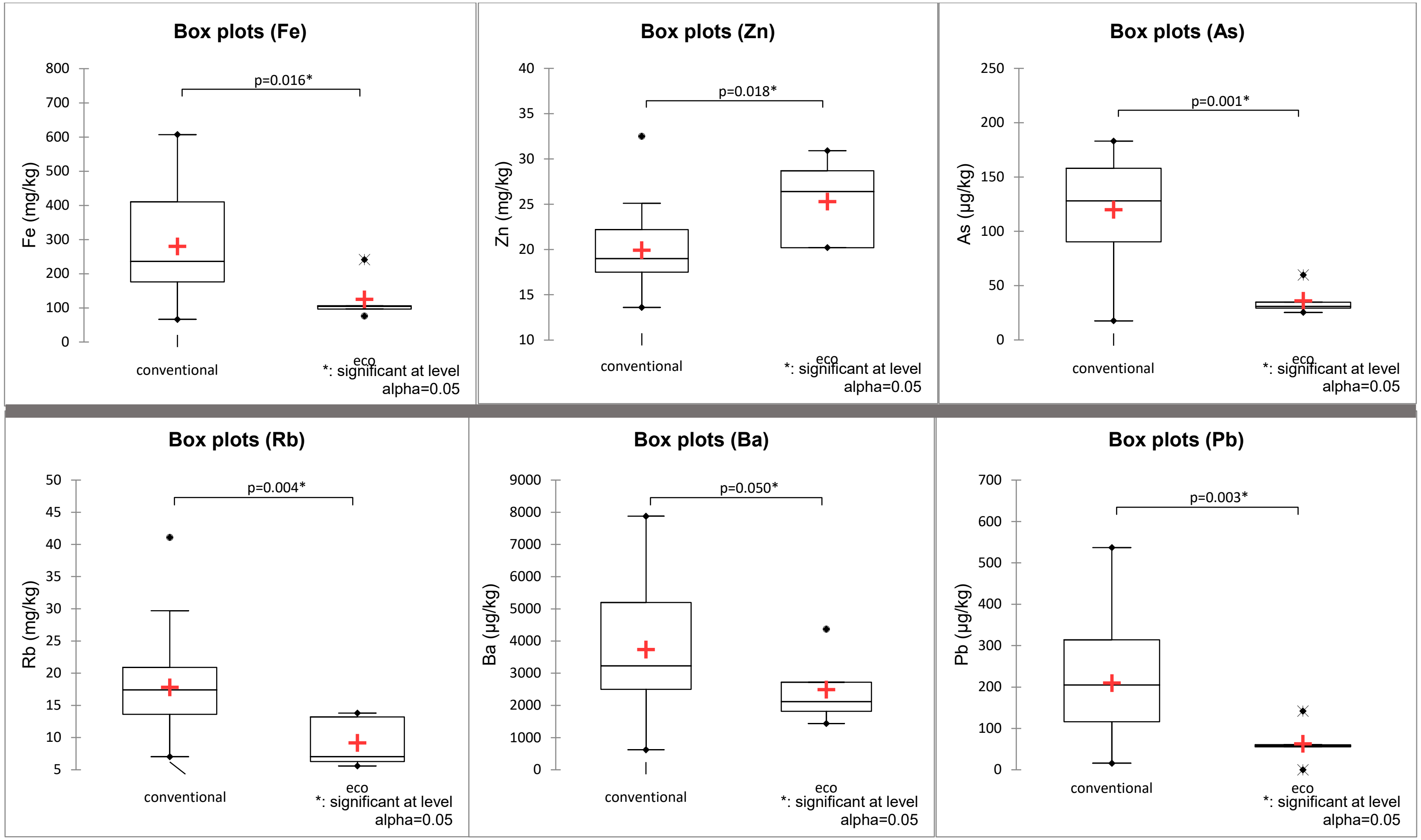
Box plots (Fe)
Fe (mg/kg)
p=0.016*
conventional
eco
*: significant at level alpha=0.05
Box plots (Zn)
Zn (mg/kg)
p=0.018*
conventional
eco
*: significant at level alpha=0.05
Box plots (As)
As (µg/kg)
p=0.001*
conventional
eco
*: significant at level alpha=0.05
Box plots (Rb)
Rb (mg/kg)
p=0.004*
conventional
eco
*: significant at level alpha=0.05
Box plots (Ba)
Ba (µg/kg)
p=0.050*
conventional
eco
*: significant at level alpha=0.05
Box plots (Pb)
Pb (µg/kg)
p=0.003*
conventional
eco
*: significant at level alpha=0.05

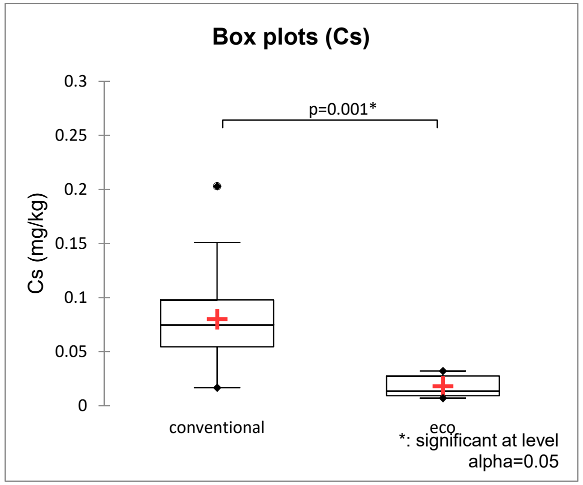

Figure S1. Box plots of elemental concentrations (Fe, Zn, As, Rb, Ba, Pb, Cs) in paprika samples by agricultural method of production. where statistical differences were observed.
